# Supplementary material for: CRISPR/Cas13 effectors have differing extents of off-target effects that limit their utility in eukaryotic cells
Source: Nucleic Acids Res. 2022 Mar 4;50(11):e65. doi: 10.1093/nar/gkac159 (PMC9226543; doi:10.1093/nar/gkac159)
Supplement: gkac159_Supplemental_File [file gkac159_supplemental_file.pdf]

SUPPLEMENTARY FIGURES

Supplementary Figure S1

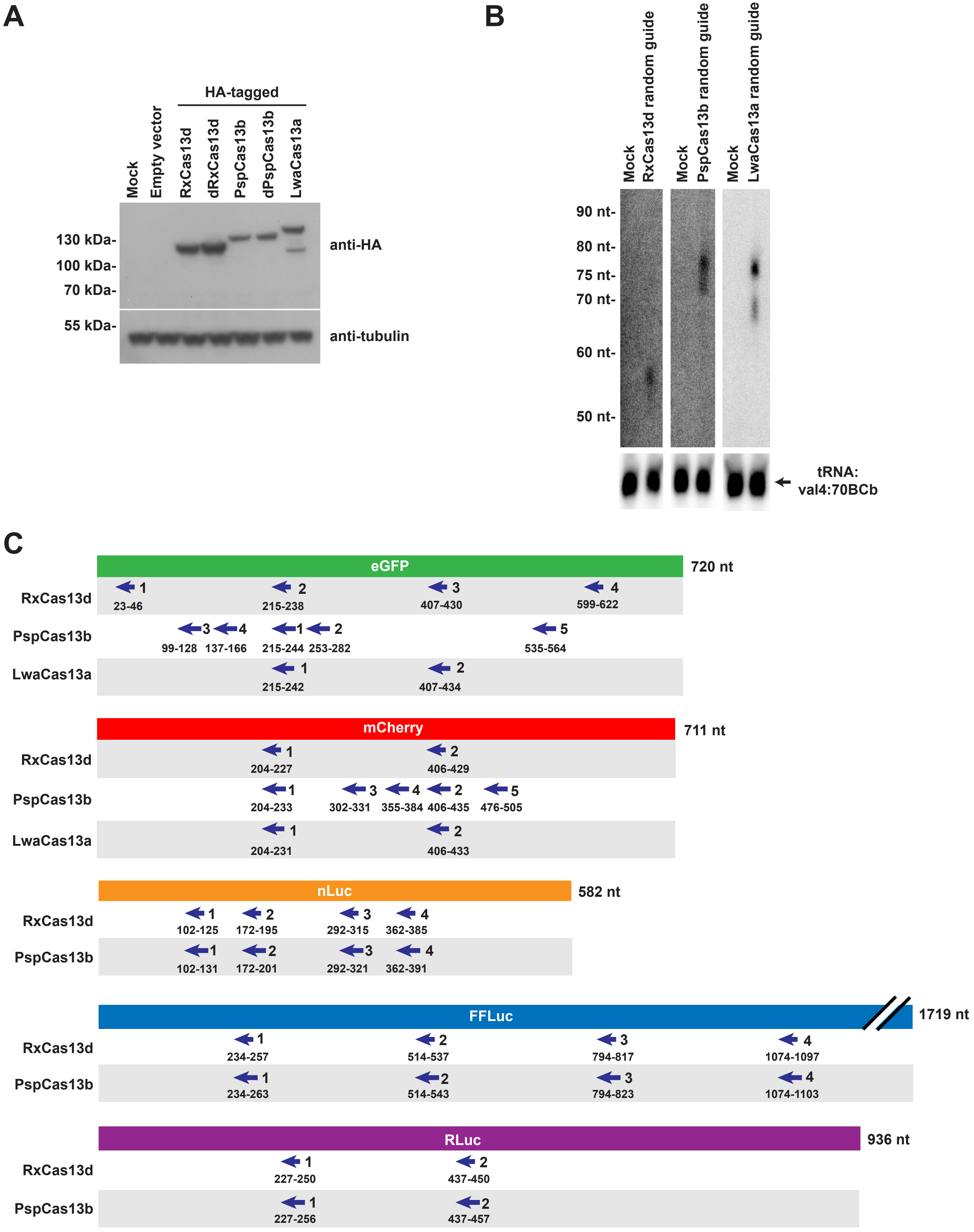

Supplementary Figure S1. *Drosophila* Cas13 and guide RNA expression plasmids. (A-B) Analogous to Figure 1A, *Drosophila* DL1 cells were co-transfected with (i) 50 ng of plasmid that expresses the indicated HA-tagged Cas13 effector as well as a random guide RNA, (ii) 225 ng of plasmid that expresses eGFP from the copper-inducible MtnA promoter, and (iii) 225 ng of plasmid that expresses mCherry from the MtnA promoter. 24 hr after transfection, $CuSO_4$ was added for 14 hr and total protein **(A)** or RNA **(B)** was then isolated. **(A)** Representative Western blot using an α-HA antibody to confirm Cas13 effector protein expression. α-Tubulin was used as a loading control. **(B)** Northern blots using 8% polyacrylamide gels (20 μg of total RNA/lane) were performed to detect guide RNA expression. Expected guide RNA lengths: RxCas13d (59 nt), PspCas13b (75 nt), LwaCas13a (71 nt). tRNA:val4:70BCb was used as a loading control. **(C)** The guide RNAs complementary to eGFP, mCherry, nanoluciferase (nLuc), firefly luciferase (FFLuc), and Renilla luciferase (RLuc) that were used in this study.

Supplementary Figure S2

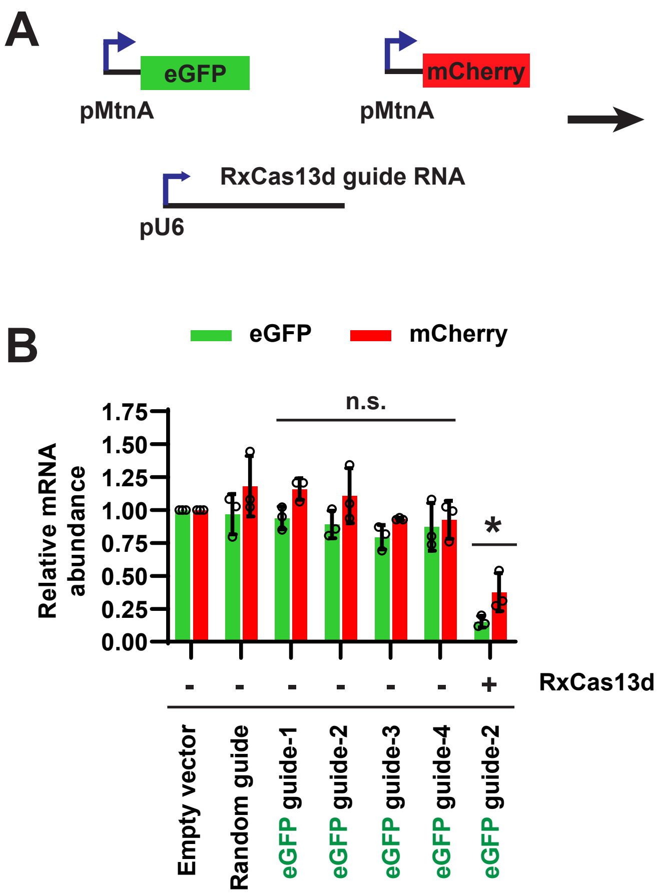

Supplementary Figure S2. Transfection of a RxCas13d guide RNA alone is insufficient to deplete target mRNAs in *Drosophila cells*. (A) *Drosophila* DL1 cells were co-transfected with (i) 50 ng of plasmid that constitutively expresses a guide RNA from the U6 promoter, (ii) 225 ng of plasmid that expresses eGFP from the copper-inducible MtnA promoter, and (iii) 225 ng of plasmid that expresses mCherry from the MtnA promoter. 24 hr after transfection, $CuSO_4$ was added and total RNA was isolated after an additional 14 hr. **(B)** RT-qPCR was used to quantify relative expression of eGFP and mCherry mRNAs. As a positive control, a plasmid expressing the RxCas13d effector as well as a guide RNA complementary to eGFP was used. Data are shown as mean ± SD, N=3. For statistical comparisons, data were compared to the random guide RNA samples. (*) $P < 0.05$. n.s., not significant.

Supplementary Figure S3

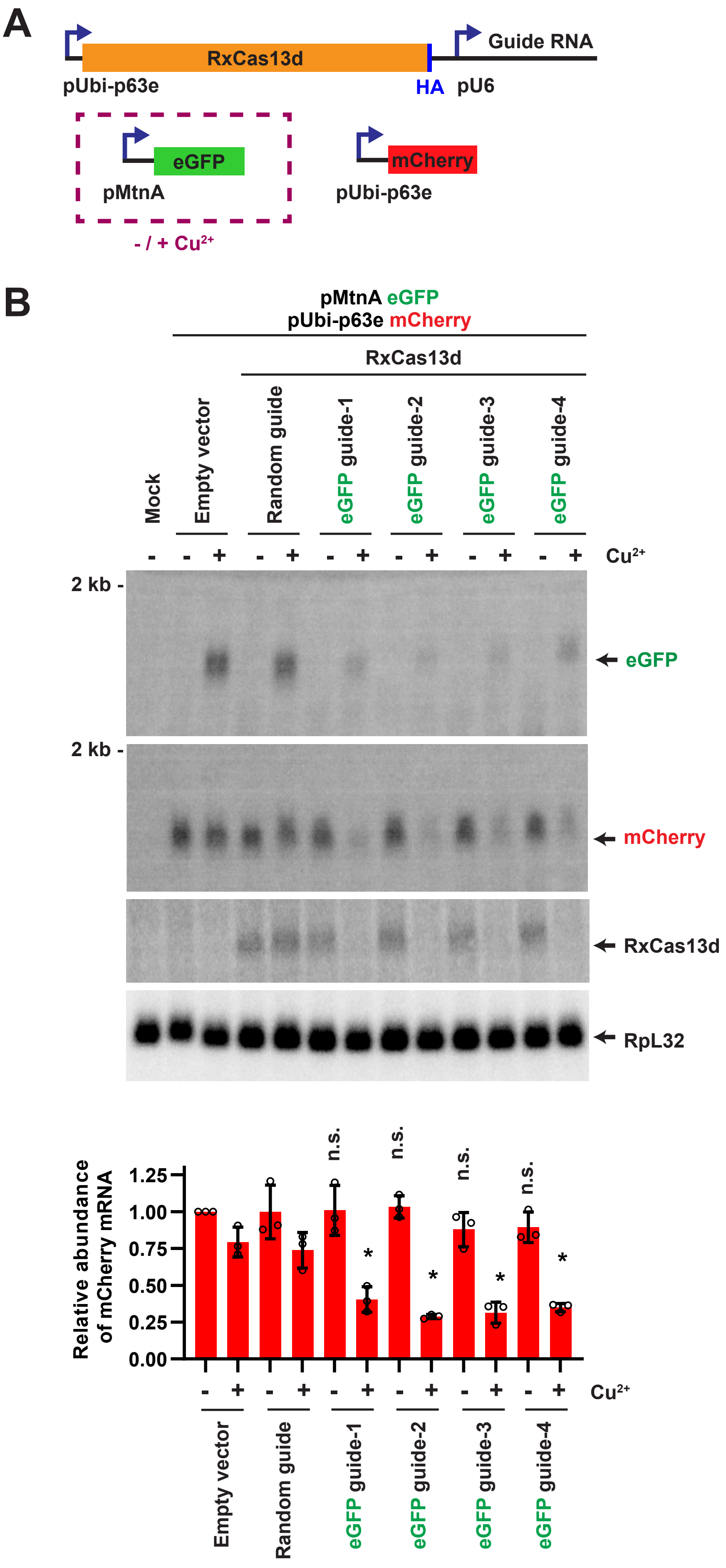

Supplementary Figure S3. RxCas13d off-target effects are dependent on transcription of the target mRNA in *Drosophila* cells. (A) *Drosophila* DL1 cells were co-transfected with (i) 50 ng of plasmid that constitutively expresses a guide RNA from the U6 promoter as well as catalytically active RxCas13d from the Ubi-p63e promoter, (ii) 225 ng of plasmid that expresses eGFP from the copper-inducible MtnA promoter, and (iii) 225 ng of plasmid that expresses mCherry from the Ubi-p63e promoter. After 24 hr, expression of eGFP was (+) or was not (-) induced by adding $CuSO_4$ for 14 hr, as indicated. **(B)** Total RNA was then isolated and Northern blots (20 μg of total RNA/lane) used to quantify the relative expression levels of eGFP, mCherry, and RxCas13d mRNAs. Representative blots are shown. mCherry mRNA expression data are shown as mean ± SD, N=3. For statistical comparisons, data were compared to the random guide RNA samples. (*) $P < 0.05$. n.s., not significant.

Supplementary Figure S4

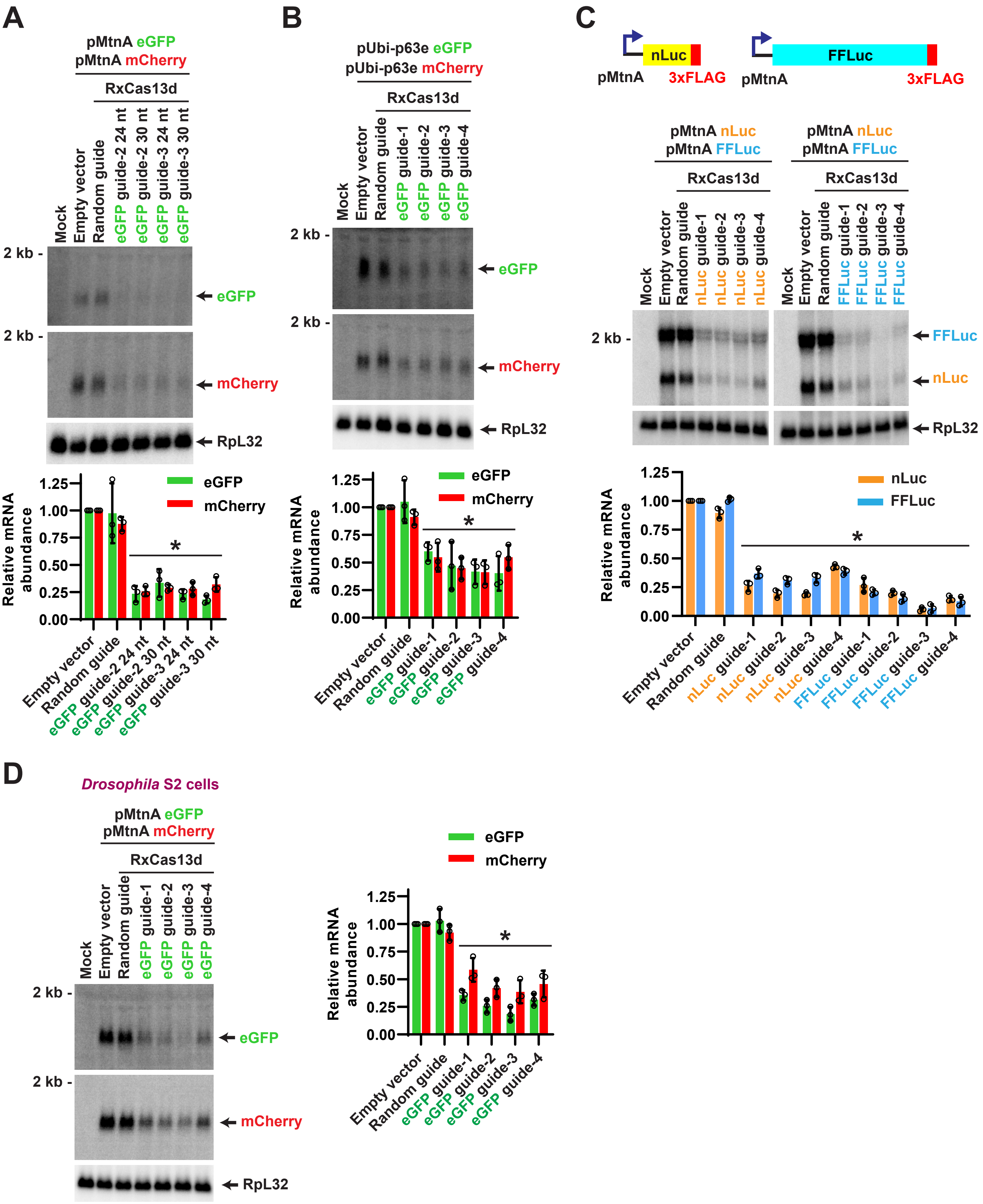

Supplementary Figure S4. Strong off-target effects of RxCas13d are observed under multiple conditions in *Drosophila* cells. (A) *Drosophila* DL1 cells were co-transfected with RxCas13d-guide RNA (50 ng), eGFP (225 ng), and mCherry (225 ng) expression plasmids as described in Figure 1A except that two different lengths (24 and 30 nt) of guide RNA spacers were tested. 24 hr after transfection, $CuSO_4$ was added and total RNA was isolated after an additional 14 hr. Northern blots (20 μg of total RNA/lane) were then performed to quantify expression of eGFP and mCherry mRNAs. **(B)** *Drosophila* DL1 cells were co-transfected as in Figure 1A except that eGFP and mCherry were expressed from the constitutive Ubi-p63e promoter (not the copper-inducible MtnA promoter). 40 hr after transfection, total RNA was isolated and Northern blots performed (20 μg of total RNA/lane). **(C)** *Drosophila* DL1 cells were co-transfected with (i) 50 ng of plasmid expressing RxCas13d and a guide RNA complementary to nLuc (left) or FFLuc (right), (ii) 225 ng of plasmid that expresses 3xFLAG tagged nLuc from the copper-inducible MtnA promoter, and (iii) 225 ng of plasmid that expresses 3xFLAG tagged FFLuc from the MtnA promoter. 24 hr after transfection, $CuSO_4$ was added and total RNA was isolated after an additional 14 hr. Northern blotting (20 μg of total RNA/lane) using a probe complementary to the 3xFLAG tag was then performed. **(D)** *Drosophila* S2 cells were co-transfected with (i) 200 ng of plasmid that expresses RxCas13d and a guide RNA complementary to eGFP, (ii) 900 ng of plasmid that expresses eGFP from the copper-inducible MtnA promoter, and (iii) 900 ng of plasmid that expresses mCherry from the MtnA promoter. 24 hr after transfection, $CuSO_4$ was added and total RNA was isolated after an additional 14 hr. Northern blotting (20 μg of total RNA/lane) was then performed. For all panels **(A-D)**, representative Northern blots are shown. ImageQuant was used to quantify the relative expression levels of the indicated mRNAs (normalized to the empty vector samples) from three independent experiments. RpL32 mRNA served as an endogenous loading control. Data are shown as mean ± SD. For statistical comparisons, data were compared to the random guide RNA samples. (∗) $P < 0.05$.

Supplementary Figure S5

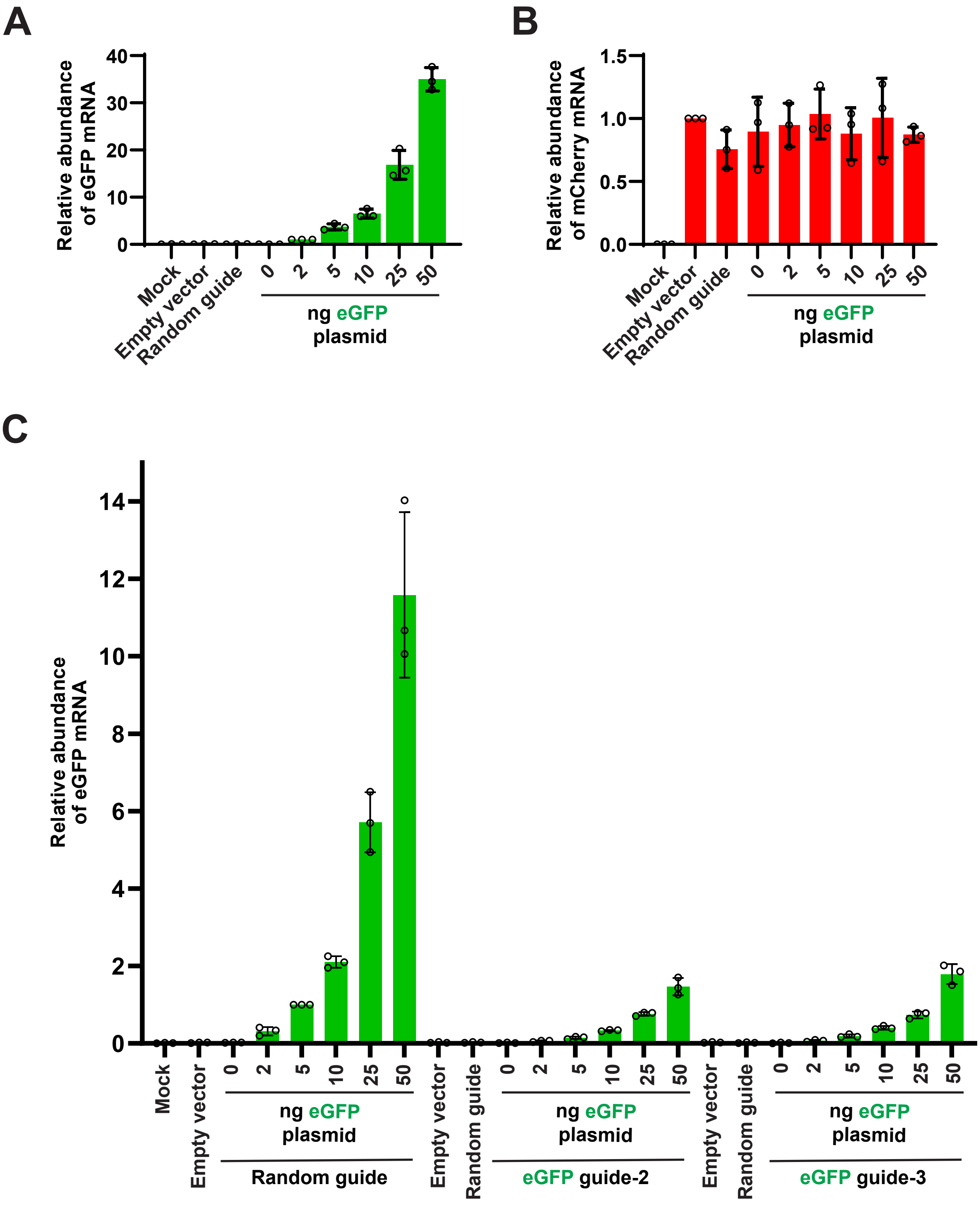

Supplementary Figure S5. Titration of eGFP target mRNA expression to characterize dose-dependent off-target effects of RxCas13d in *Drosophila* cells. (A-B) *Drosophila* DL1 cells were transfected with increasing amounts of copper-inducible eGFP expression plasmid (0, 2, 5, 10, 25, or 50 ng) along with a constant amount (225 ng) of copper-inducible mCherry expression plasmid. Empty vector (pUb-3xFLAG MCS (No BsmBI) plasmid) was added as needed so that 500 ng DNA was transfected in all samples. 24 hr after transfection, $CuSO_4$ was added for 14 hr and total RNA then isolated. RT-qPCR was used to quantify relative expression of eGFP **(A)** and mCherry **(B)** mRNAs. eGFP data were normalized to the ‘eGFP 2 ng’ sample, while mCherry data were normalized to the ‘empty vector’ samples. Data are shown as mean ± SD, N=3. **(C)** Same RT-qPCR data as in Figure 3B, but with all eGFP expression levels normalized to the ‘eGFP 5 ng - Random guide RNA’ sample. Data are shown as mean ± SD, N=3.

Supplementary Figure S6

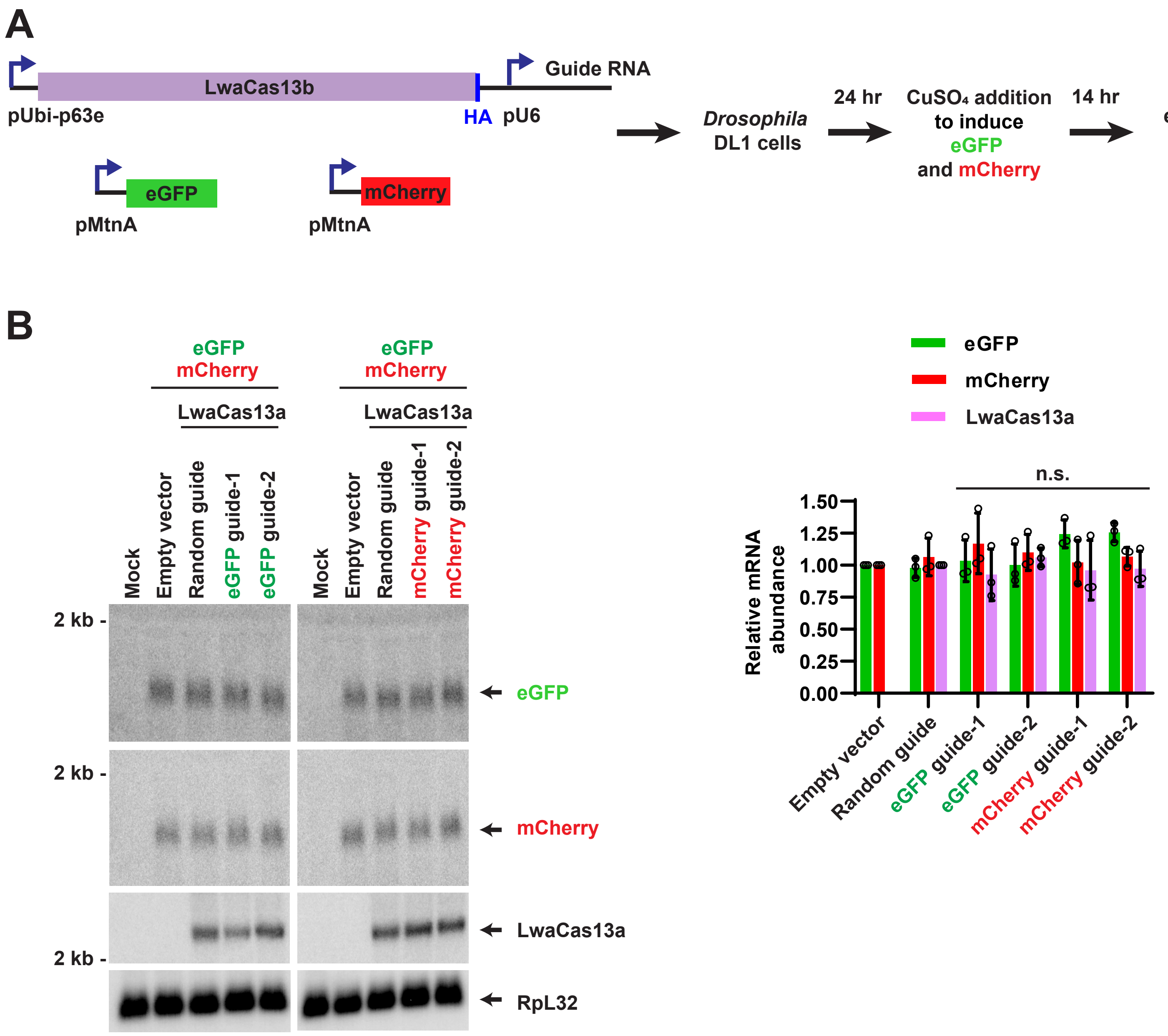

Supplementary Figure S6. LwaCas13a failed to deplete target RNAs in *Drosophila* cells. **(A)** Co-transfection assay analogous to Figure 1A except that LwaCas13a rather than RxCas13d was examined. *Drosophila* DL1 cells were co-transfected with (i) 50 ng of plasmid that constitutively expresses a guide RNA from the U6 promoter as well as HA-tagged LwaCas13a from the Ubi-p63e promoter, (ii) 225 ng of plasmid that expresses eGFP from the copper-inducible MtnA promoter, and (iii) 225 ng of plasmid that expresses mCherry from the MtnA promoter. 24 hr after transfection, $CuSO_4$ was added and total RNA was isolated after an additional 14 hr. Northern blots were then performed. **(B)** Representative Northern blots (20 μg of total RNA/lane) are shown from experiments in which guide RNAs complementary to eGFP (left) or mCherry (right) were used. ImageQuant was used to quantify the relative expression levels of eGFP, mCherry, and LwaCas13a mRNAs from three independent experiments. eGFP and mCherry mRNA expression was normalized to the empty vector samples, while LwaCas13a mRNA expression was normalized to the random guide RNA samples. Data are shown as mean ± SD. n.s., not significant.

Supplementary Figure S7

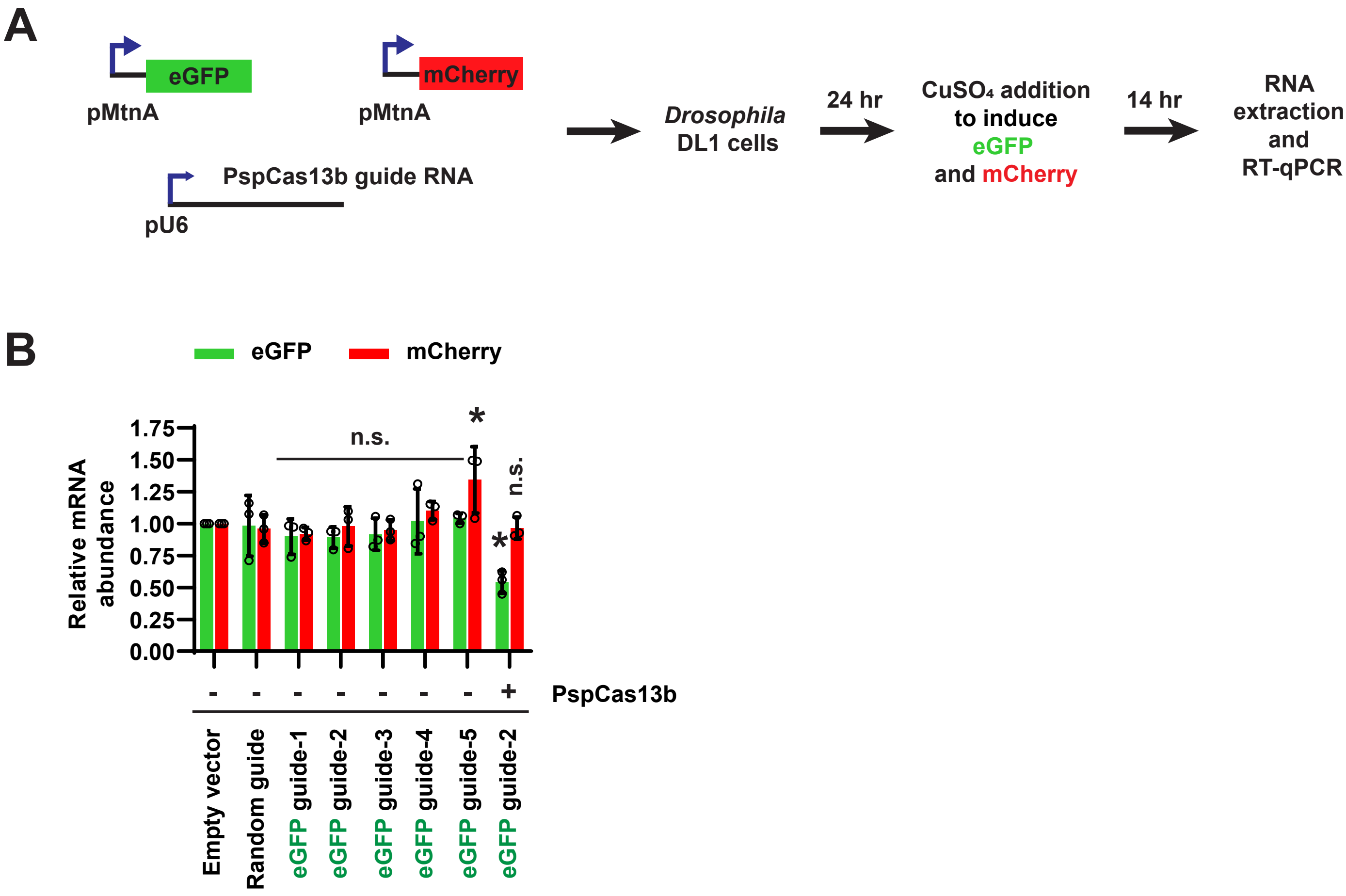

Supplementary Figure S7. Transfection of a PspCas13b guide RNA alone is insufficient to deplete target mRNAs in *Drosophila cells*. (A) *Drosophila* DL1 cells were co-transfected with (i) 50 ng of plasmid that constitutively expresses a guide RNA from the U6 promoter, (ii) 225 ng of plasmid that expresses eGFP from the copper-inducible MtnA promoter, and (iii) 225 ng of plasmid that expresses mCherry from the MtnA promoter. 24 hr after transfection, $CuSO_4$ was added and total RNA was isolated after an additional 14 hr. **(B)** RT-qPCR was used to quantify relative expression of eGFP and mCherry mRNAs. As a positive control, a plasmid expressing the PspCas13b effector as well as a guide RNA complementary to eGFP was used. Data are shown as mean ± SD, N=3. For statistical comparisons, data were compared to the random guide RNA samples. (*) $P < 0.05$. n.s., not significant.

Supplementary Figure S8

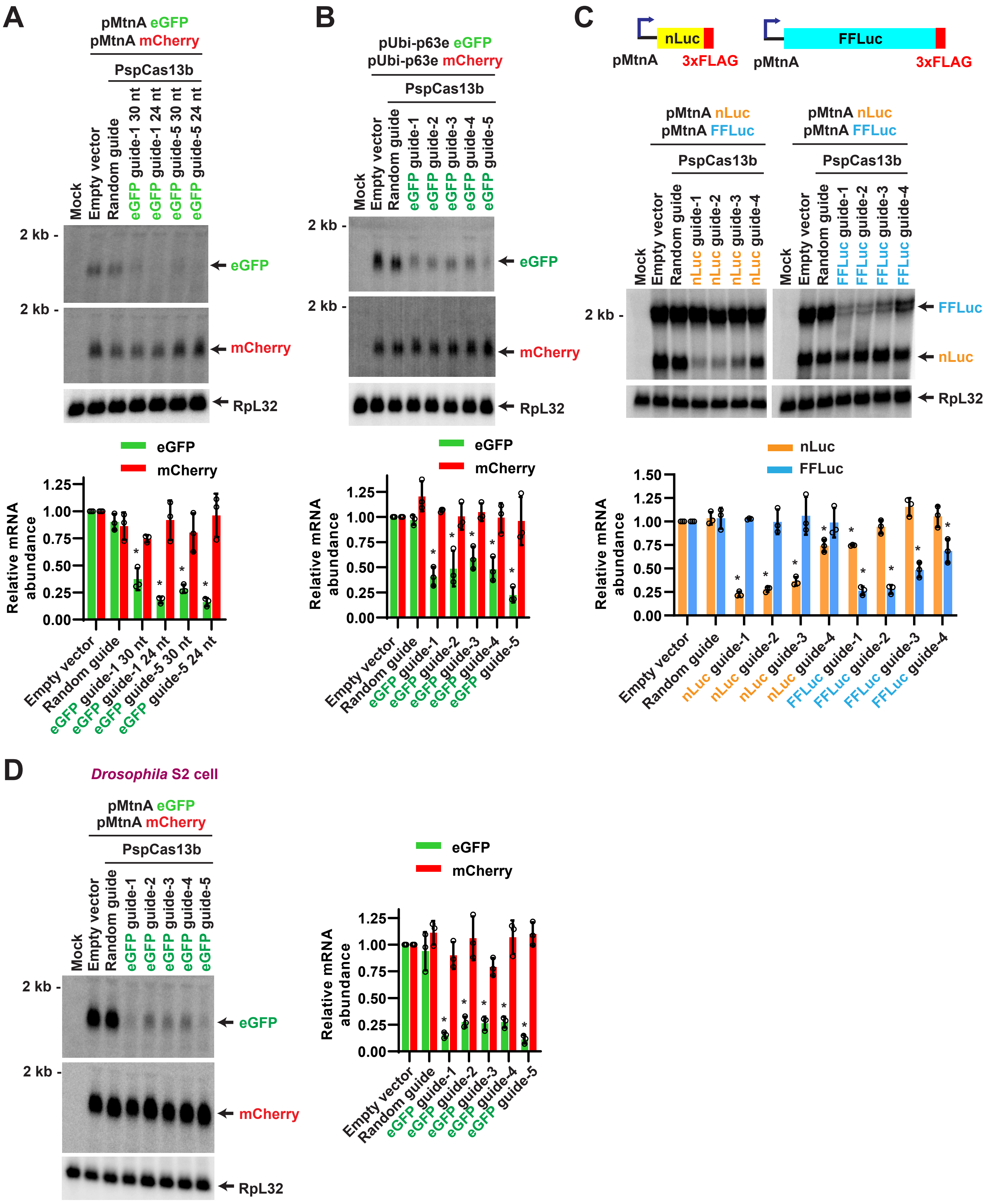

Supplementary Figure S8. PspCas13b has good specificity under multiple conditions in *Drosophila* cells. These experiments are the same as those performed in Supplementary Figure S4 except that PspCas13b rather than RxCas13d was examined. **(A)** *Drosophila* DL1 cells were co-transfected with PspCas13b-guide RNA (50 ng), eGFP (225 ng), and mCherry (225 ng) expression plasmids. Two different lengths (24 and 30 nt) of guide RNA spacers were tested. 24 hr after transfection, $CuSO_4$ was added and total RNA was isolated after an additional 14 hr. Northern blots (20 μg of total RNA/lane) were then performed to quantify expression of eGFP and mCherry mRNAs. **(B)** *Drosophila* DL1 cells were co-transfected as in Figure 4A except that the eGFP and mCherry expression plasmids were driven by the constitutive Ubi-p63e promoter (not the copper-inducible MtnA promoter). 40 hr after transfection, total RNA was isolated and Northern blots (20 μg of total RNA/lane) performed. **(C)** *Drosophila* DL1 cells were co-transfected with (i) 50 ng of plasmid expressing PspCas13b and a guide RNA complementary to nLuc (left) or FFLuc (right), (ii) 225 ng of plasmid that expresses 3xFLAG tagged nLuc from the copper-inducible MtnA promoter, and (iii) 225 ng of plasmid that expresses 3xFLAG tagged FFLuc from the MtnA promoter. 24 hr after transfection, $CuSO_4$ was added and total RNA was isolated after an additional 14 hr. Northern blotting (20 μg of total RNA/lane) using a probe complementary to the 3xFLAG tag was then performed. **(D)** *Drosophila* S2 cells were co-transfected with (i) 500 ng of plasmid that expresses PspCas13b and a guide RNA complementary to eGFP, (ii) 750 ng of plasmid that expresses eGFP from the copper-inducible MtnA promoter, and (iii) 750 ng of plasmid that expresses mCherry from the MtnA promoter. 24 hr after transfection, $CuSO_4$ was added and total RNA was isolated after an additional 14 hr. Northern blotting (20 μg of total RNA/lane) was then performed. For all panels **(A-D)**, representative Northern blots are shown. ImageQuant was used to quantify the relative expression levels of the indicated mRNAs (normalized to the empty vector samples) from three independent experiments. RpL32 mRNA served as an endogenous loading control. Data are shown as mean ± SD. For statistical comparisons, data were compared to the random guide RNA samples. (*) $P < 0.05$.

Supplementary Figure S9

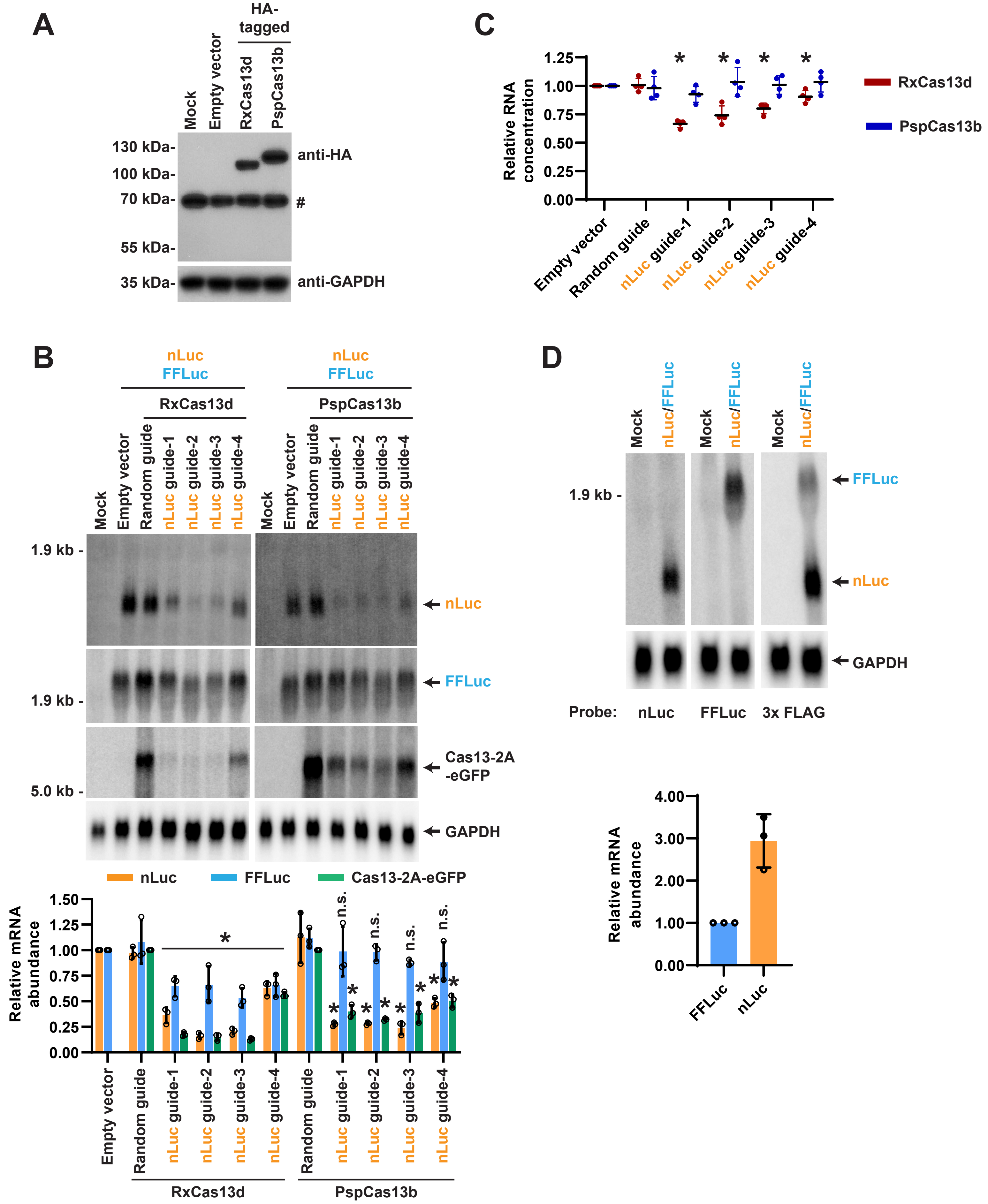

Supplementary Figure S9. Specificity of RxCas13d and PspCas13b in HeLa cells when nLuc and FFLuc were co-transfected. (A-C) Analogous to Figure 6A, HeLa cells were co-transfected with (i) 300 ng of plasmid that expresses HA-tagged Cas13 protein followed by a 2A peptide and eGFP, (ii) 200 ng of plasmid that expresses a guide RNA, (iii) 250 ng of plasmid that expresses 3xFLAG-tagged nLuc, and (iv) 250 ng of plasmid that expresses 3xFLAG-tagged FFLuc. 48 hr after transfection, total protein **(A)** or RNA **(B-C)** was isolated. **(A)** A representative Western blot using an α-HA antibody to confirm Cas13 protein expression (Note: random guide RNA plasmid was co-transfected). α-GAPDH was used as a loading control. # denotes non-specific band. **(B)** Representative Northern blots (20 μg of total RNA/lane) are shown. ImageQuant was used to quantify the relative expression levels of nLuc, FFLuc, and Cas13-2A-eGFP mRNAs. nLuc and FFLuc mRNA expression was normalized to the empty vector (pBEVY-L) samples, while Cas13-2A-eGFP mRNA expression was normalized to the random guide RNA samples. GAPDH mRNA served as an endogenous loading control. Data are shown as mean ± SD, N=3. For statistical comparisons, data were compared to the random guide RNA samples. ($*$) $P < 0.05$. **(C)** Relative RNA concentrations obtained from the co-transfection assays when the RxCas13d or PspCas13b expression plasmids were used. Data are normalized to the empty vector samples and shown as mean ± SD, N=4. ($*$) $P < 0.05$. **(D)** HeLa cells were co-transfected with (i) 250 ng of plasmid that expresses 3xFLAG-tagged nLuc, (ii) 250 ng of plasmid that expresses 3xFLAG-tagged FFLuc, and (iii) 500 ng of empty vector (pBEVY-L). 48 hr after transfection, total RNA was isolated. Northern blots (20 μg of total RNA/lane) were then performed using probes complementary to nLuc, FFLuc, or the 3xFLAG that detects both nLuc and FFLuc mRNAs. ImageQuant was used to quantify the relative expression levels of nLuc and FFLuc mRNAs that were detected using the 3x FLAG probe. nLuc expression was normalized to FFLuc expression. Data are shown as mean ± SD, N=3.

Supplementary Figure S10

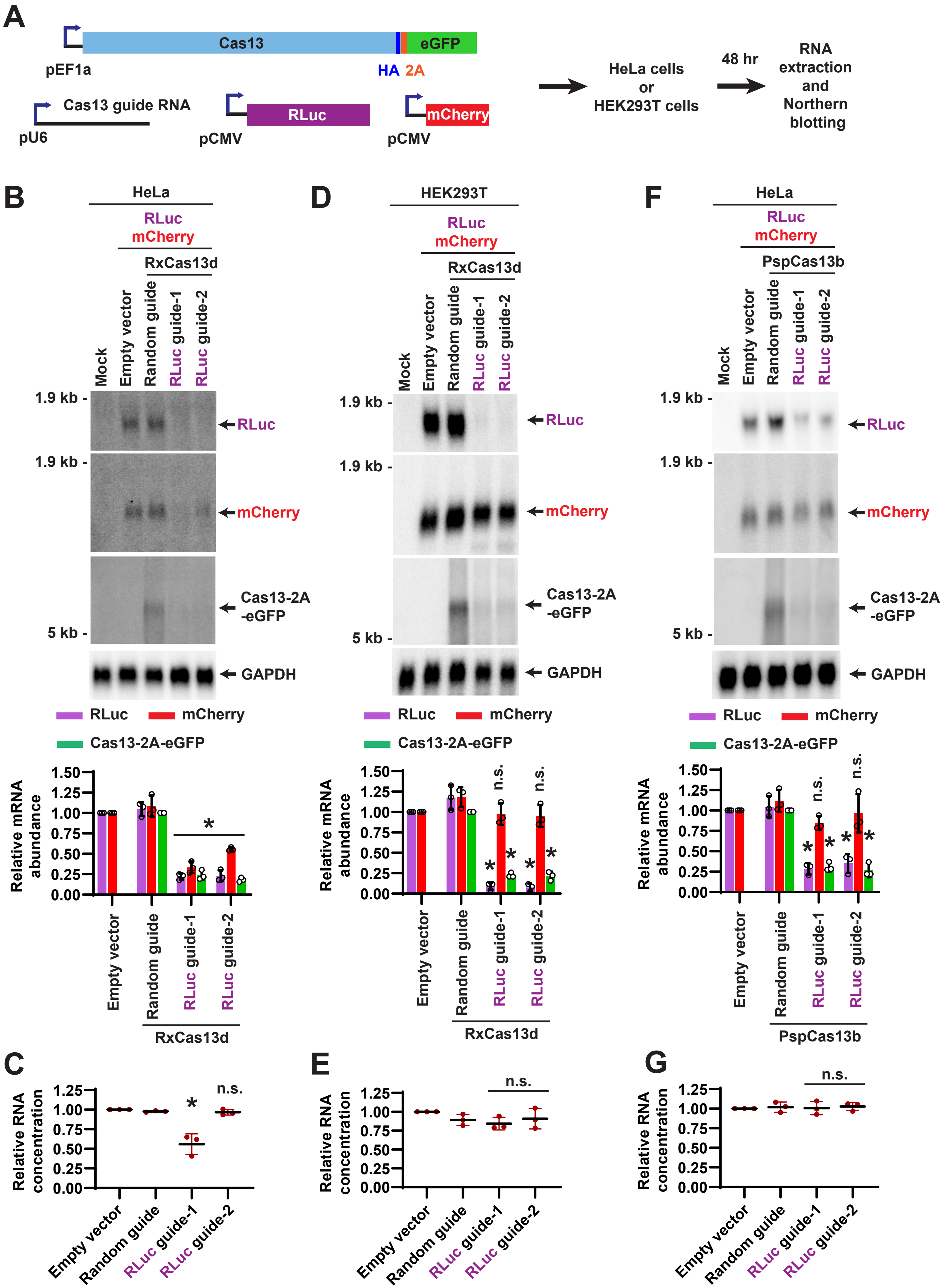

Supplementary Figure S10. Specificity of Cas13 effectors when RLuc and mCherry were co-transfected into different human cell lines. (A) HeLa or HEK293T cells were co-transfected with (i) 300 ng of plasmid that constitutively expresses HA-tagged Cas13 protein followed by a 2A peptide and eGFP, (ii) 200 ng of plasmid that expresses a guide RNA, (iii) 250 ng of plasmid that expresses Renilla luciferase (RLuc), and (iv) 250 ng of plasmid that expresses mCherry. 48 hr after transfection, total RNA was isolated and Northern blots performed. **(B-E)** RxCas13d and guide RNAs complementary to RLuc were employed in the co-transfection assay in HeLa **(B,C)** or HEK293T cells **(D,E)**. **(B,D)** Representative Northern blots (20 μg of total RNA/lane) are shown. ImageQuant was used to quantify the relative expression levels of RLuc, mCherry, and Cas13-2A-eGFP mRNAs. RLuc and mCherry mRNA expression was normalized to the empty vector (pBEVY-L) samples, while Cas13-2A-eGFP mRNA expression was normalized to the random guide RNA samples. GAPDH mRNA served as an endogenous loading control. Data are shown as mean ± SD, N=3. For statistical comparisons, data were compared to the random guide RNA samples. (∗) $P < 0.05$. **(C,E)** Relative RNA concentrations obtained from the co-transfection assays in HeLa **(C)** and HEK293T **(E)** when the RxCas13d expression plasmid was used. Data are normalized to the empty vector samples and shown as mean ± SD, N=3. (∗) $P < 0.05$. **(F-G)** PspCas13b and guide RNAs complementary to RLuc were employed in the co-transfection assay in HeLa cells as diagrammed in A. **(F)** Representative Northern blots (20 μg of total RNA/lane) are shown. ImageQuant was used to quantify the relative expression levels of RLuc, mCherry, and Cas13-2A-eGFP mRNAs. RLuc and mCherry mRNA expression was normalized to the empty vector (pBEVY-L) samples, while Cas13-2A-eGFP mRNA expression was normalized to the random guide RNA samples. GAPDH mRNA served as an endogenous loading control. Data are shown as mean ± SD, N=3. For statistical comparisons, data were compared to the random guide RNA samples. (∗) $P < 0.05$. **(G)** Relative RNA concentrations obtained from the co-transfection assays. Data are normalized to the empty vector samples and shown as mean ± SD, N=3. No significant changes were observed.

Supplementary Figure S11

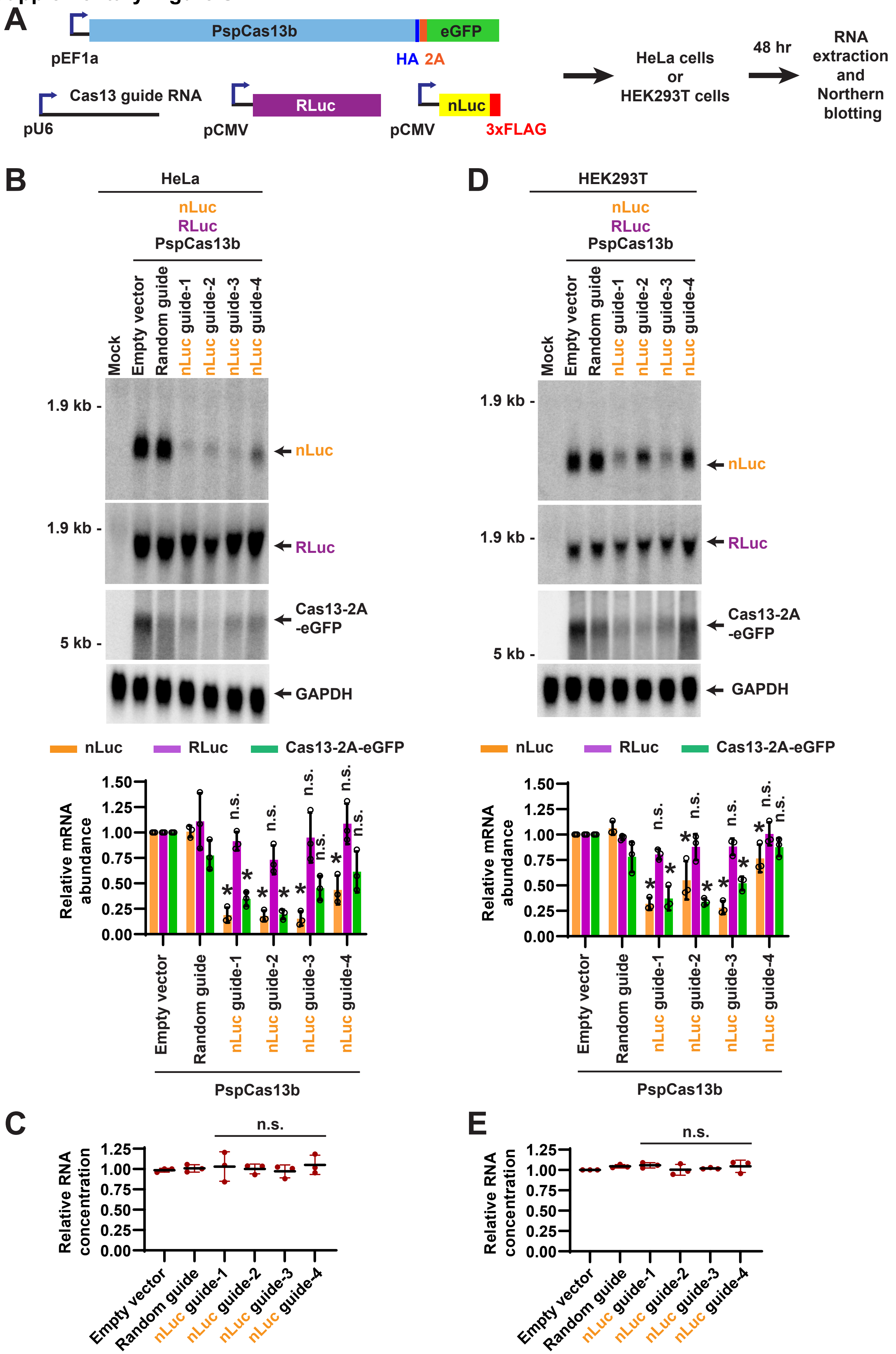

Supplementary Figure S11. Specificity of PspCas13b when RLuc and nLuc were co-transfected into HeLa or HEK293T cells. (A) HeLa or HEK293T cells were co-transfected with (i) 300 ng of plasmid that constitutively expresses HA-tagged PspCas13b followed by a 2A peptide and eGFP, (ii) 200 ng of plasmid that expresses a guide RNA, (iii) 250 ng of plasmid that expresses Renilla luciferase (RLuc), and (iv) 250 ng of plasmid that expresses 3xFLAG-tagged nLuc. 48 hr after transfection, total RNA was isolated and Northern blots performed. **(B-E)** Guide RNAs complementary to nLuc were employed in the co-transfection assay in HeLa **(B-C)** or HEK293T cells **(D-E)**. **(B, D)** Representative Northern blots (20 μg of total RNA/lane) are shown. ImageQuant was used to quantify the relative expression levels of RLuc, nLuc, and Cas13-2A-eGFP mRNAs. RLuc and nLuc mRNA expression was normalized to the empty vector (pBEVY-L) samples, while Cas13-2A-eGFP mRNA expression was normalized to the random guide RNA samples. GAPDH mRNA served as an endogenous loading control. Data are shown as mean ± SD, N=3. For statistical comparisons, data were compared to the random guide RNA samples. (*) $P < 0.05$. **(C, E)** Relative RNA concentrations obtained from the co-transfection assays. Data are normalized to the empty vector samples and shown as mean ± SD, N=3. (*) $P < 0.05$. n.s., not significant.

SUPPLEMENTARY TABLES

Supplementary Table S1

Guide RNAs used in *Drosophila* cells

Cas protein	Guide RNA	Forward primer	Reverse primer
RxCas13d	Random guide (DL1)	gaaacCGAGGGCGACTTAACCTTAGGTt	aaaaaACCTAAGGTTAAGTCGCCCTCGg
RxCas13d	Random guide (S2)	gaaacGCAGGGTTTTCCCAGTCACGACGTt	aaaaaACGTCGTGACTGGGAAAACCCTGCg
RxCas13d	eGFP guide-1	GAAACGGATGGGCACCACCCCGGTGAACAT	AAAAATGTTCACCGGGGTGGTGCCCATCCG
RxCas13d	eGFP guide-2	GAAACTCATGTGGTCGGGGTAGCGGCTGAT	AAAAATCAGCCGCTACCCCGACCACATGAG
RxCas13d	eGFP guide-3	GAAACACTCCAGCTTGTGCCCCAGGATGTT	AAAAAACATCCTGGGGCACAAGCTGGAGTG
RxCas13d	eGFP guide-4	GAAACGGGCGGACTGGGTGCTCAGGTAGTT	AAAAAACTACCTGAGCACCCAGTCCGCCCG
RxCas13d	mCherry guide-1	gaaacgccttggagccgtacatgaactgat	aaaaatcagttcatgtacggctccaaggcg
RxCas13d	mCherry guide-2	gaaaccttctgcattacggggccgtcggat	aaaaatccgacggccccgtaatgcagaagg
RxCas13d	Laccase2 BSJ guide	GAAACGGTAACCGCTTCCAGCGGTTAGGGT	AAAAAGGGGAGCTCAGCAAAGGTACCAAAG
RxCas13d	Laccase2 Exon 2 guide	GAAACGGTAACCGCTTCCAGCGGTTAGGGT	AAAAACCCTAACCGCTGGAAGCGGTTACCG
RxCas13d	Laccase2 Exon 3 guide	GAAACGTCCATCTAGTTTCTGTAGACCGGT	AAAAACCGGTCTACAGAAACTAGATGGACG
RxCas13d	nLuc guide-1	GAAACGGAGTTACGGACACCCCGAGATTCT	AAAAAGAATCTCGGGGTGTCCGTAACTCCG
RxCas13d	nLuc guide-2	GAAACTTCATACGGGATGATGACATGGATT	AAAAAATCCATGTCATCATCCCGTATGAAG
RxCas13d	nLuc guide-3	GAAACCGTAACCCCGTCGATTACCAGTGTT	AAAAAACACTGGTAATCGACGGGGTTACGG
RxCas13d	nLuc guide-4	GAAACCAGTGATCTTTTTGCCGTCGAACAT	AAAAATGTTCGACGGCAAAAAGATCACTGG
RxCas13d	FFLuc guide-1	GAAACaagctattctcgctgcacaccacgT	AAAAAcgtggtgtgcagcgagaatagcttG
RxCas13d	FFLuc guide-2	GAAACgtactcgttgaagccgggtggcaaT	AAAAAttgccacccggcttcaacgagtacG
RxCas13d	FFLuc guide-3	GAAACatagctcctcctcgaagcggtacaT	AAAAAtgtaccgcttcgaggaggagctatG
RxCas13d	FFLuc guide-4	GAAACaccaccttgcctactgcgccaggcT	AAAAAgcctggcgcagtaggcaaggtggtG
RxCas13d	eGFP guide-2 30 nt	GAAACGCTGCTTCATGTGGTCGGGGTAGCGGCTGAT	AAAAATCAGCCGCTACCCCGACCACATGAAGCAGCG
RxCas13d	eGFP guide-3 30 nt	GAAACAGTTGTACTCCAGCTTGTGCCCCAGGATGTT	AAAAAACATCCTGGGGCACAAGCTGGAGTACAACTG
PspCas13b	Random guide (DL1)	cttcggagacggtCGAGGGCGACTTAACCTTAGGT	caacACCTAAGGTTAAGTCGCCCTCGaccgtctcc
PspCas13b	Random guide (S2)	cttcgGCAGGGTTTTCCCAGTCACGACGTTGTAAA	caacTTTACAACGTCGTGACTGGGAAAACCCTGCc
PspCas13b	eGFP guide-1	cttcgGCTGCTTCATGTGGTCGGGGTAGCGGCTGA	caacTCAGCCGCTACCCCGACCACATGAAGCAGCc
PspCas13b	eGFP guide-2	cttcgGACGTAGCCTTCGGGCATGGCGGACTTGAA	caacTTCAAGTCCGCCATGCCCGAAGGCTACGTCc
PspCas13b	eGFP guide-3	cttcgAGCTTGCCGTAGGTGGCATCGCCCTCGCCC	caacGGGCGAGGGCGATGCCACCTACGGCAAGCTc
PspCas13b	eGFP guide-4	cttcgCGGGCAGCTTGCCGGTGGTGCAGATGAACT	caacAGTTCATCTGCACCACCGGCAAGCTGCCCGc
PspCas13b	eGFP guide-5	cttcgGGGGGTGTTCTGCTGGTAGTGGTCGGCGAG	caacCTCGCCGACCACTACCAGCAGAACACCCCCc
PspCas13b	mCherry guide-1	cttcgacgtaggccttggagccgtacatgaactga	caactcagttcatgtacggctccaaggcctacgtc
PspCas13b	mCherry guide-2	cttcgggtcttcttctgcattacggggccgtcgga	caactccgacggccccgtaatgcagaagaagaccc
PspCas13b	mCherry guide-3	cttcgtcaccacgccgccgtcctcgaagttcatca	caactgatgaacttcgaggacggcggcgtggtgac
PspCas13b	mCherry guide-4	cttcgcttcaccttgtagatgaactcgccgtcctg	caaccaggacggcgagttcatctacaaggtgaagc
PspCas13b	mCherry guide-5	cttcgtctgcttgatctcgcccttcagggcgccgt	caacacggcgccctgaagggcgagatcaagcagac
PspCas13b	Laccase2 BSJ guide	cttcgGATTTTGGTACCTTTGCTGAGCTCCCCGGG	caacCCCGGGGAGCTCAGCAAAGGTACCAAAATCc
PspCas13b	Laccase2 Exon 2 guide	cttcgTTAGGTAACCGCTTCCAGCGGTTAGGGCTG	caacCAGCCCTAACCGCTGGAAGCGGTTACCTAAc
PspCas13b	Laccase2 Exon 3 guide	cttcgAGAGTCCATCTAGTTTCTGTAGACCGGTAT	caacATACCGGTCTACAGAAACTAGATGGACTCTc
PspCas13b	nLuc guide-1	cttcgTGGATCGGAGTTACGGACACCCCGAGATTC	caacGAATCTCGGGGTGTCCGTAACTCCGATCCAc
PspCas13b	nLuc guide-2	cttcgCAGACCTTCATACGGGATGATGACATGGAT	CaacATCCATGTCATCATCCCGTATGAAGGTCTGc
PspCas13b	nLuc guide-3	cttcgGTTCGGCGTAACCCCGTCGATTACCAGTGT	caacACACTGGTAATCGACGGGGTTACGCCGAACc
PspCas13b	nLuc guide-4	cttcgCTGTTACAGTGATCTTTTTGCCGTCGAACA	caacTGTTCGACGGCAAAAAGATCACTGTAACAGc
PspCas13b	FFLuc guide-1	cttcgaactgcaagctattctcgctgcacaccacg	caaccgtggtgtgcagcgagaatagcttgcagttc
PspCas13b	FFLuc guide-2	cttcggaagtcgtactcgttgaagccgggtggcaa	Caacttgccacccggcttcaacgagtacgacttcc
PspCas13b	FFLuc guide-3	cttcggcaagaatagctcctcctcgaagcggtaca	caactgtaccgcttcgaggaggagctattcttgcc
PspCas13b	FFLuc guide-4	cttcgaagggcaccaccttgcctactgcgccaggc	caacgcctggcgcagtaggcaaggtggtgcccttc
PspCas13b	eGFP guide-1 24 nt	cttcgTCATGTGGTCGGGGTAGCGGCTGA	caacTCAGCCGCTACCCCGACCACATGAc
PspCas13b	eGFP guide-5 24 nt	cttcgACTCCAGCTTGTGCCCCAGGATGT	caacACATCCTGGGGCACAAGCTGGAGTc
LwaCas13a	eGFP guide-1	aaaacTGCTTCATGTGGTCGGGGTAGCGGCTGAt	aaaaaTCAGCCGCTACCCCGACCACATGAAGCAg
LwaCas13a	eGFP guide-2	aaaacTTGTACTCCAGCTTGTGCCCCAGGATGTt	aaaaaACATCCTGGGGCACAAGCTGGAGTACAAg
LwaCas13a	mCherry guide-1	aaaacgtaggccttggagccgtacatgaactgat	aaaaatcagttcatgtacggctccaaggcctacg
LwaCas13a	mCherry guide-2	aaaactcttcttctgcattacggggccgtcggat	aaaaatccgacggccccgtaatgcagaagaagag

Supplementary Table S2
Guide RNAs used in human cells

Cas protein	Guide RNA	Forward primer	Reverse primer
RxCas13d	Random guide	aaaccGCAGGGTTTTCCCAGTCACGACGTt	aaaaaACGTCGTGACTGGGAAAACCCTGCg
RxCas13d	nLuc guide-1	AAACCGGAGTTACGGACACCCCGAGATTCT	AAAAAGAATCTCGGGGTGTCCGTAACTCCG
RxCas13d	nLuc guide-2	AAACCTTCATACGGGATGATGACATGGATT	AAAAAATCCATGTCATCATCCCGTATGAAG
RxCas13d	nLuc guide-3	AAACCCGTAACCCCGTCGATTACCAGTGTT	AAAAAACACTGGTAATCGACGGGGTTACGG
RxCas13d	nLuc guide-4	AAACCCAGTGATCTTTTTGCCGTCGAACAT	AAAAATGTTCGACGGCAAAAAGATCACTGG
RxCas13d	FFLuc guide-1	AAACCaagctattctcgctgcacaccacgT	AAAAAcgtggtgtgcagcgagaatagcttG
RxCas13d	FFLuc guide-2	AAACCgtactcgttgaagccgggtggcaaT	AAAAAttgccacccggcttcaacgagtacG
RxCas13d	FFLuc guide-3	AAACCatagctcctcctcgaagcggtacaT	AAAAAtgtaccgcttcgaggaggagctatG
RxCas13d	FFLuc guide-4	AAACCaccaccttgcctactgcgccaggcT	AAAAAgcctggcgcagtaggcaaggtggtG
RxCas13d	RLuc guide-1	AAACCacttacccattccgatcagatcagT	AAAAActgatctgatcggaatgggtaagtG
RxCas13d	RLuc guide-2	AAACCcccaggactcgatcacgtccacgaT	AAAAAtcgtggacgtgatcgagtcctgggG
PspCas13b	Random guide	caccgGCAGGGTTTTCCCAGTCACGACGTTGTAAA	caacTTTACAACGTCGTGACTGGGAAAACCCTGCc
PspCas13b	nLuc guide-1	caccgTGGATCGGAGTTACGGACACCCCGAGATTC	caacGAATCTCGGGGTGTCCGTAACTCCGATCCAc
PspCas13b	nLuc guide-2	caccgCAGACCTTCATACGGGATGATGACATGGAT	CaacATCCATGTCATCATCCCGTATGAAGGTCTGc
PspCas13b	nLuc guide-3	caccgGTTCGGCGTAACCCCGTCGATTACCAGTGT	caacACACTGGTAATCGACGGGGTTACGCCGAACc
PspCas13b	nLuc guide-4	caccgCTGTTACAGTGATCTTTTTGCCGTCGAACA	caacTGTTCGACGGCAAAAAGATCACTGTAACAGc
PspCas13b	FFLuc guide-1	caccgaactgcaagctattctcgctgcacaccacg	caaccgtggtgtgcagcgagaatagcttgcagttc
PspCas13b	FFLuc guide-2	caccggaagtcgtactcgttgaagccgggtggcaa	Caacttgccacccggcttcaacgagtacgacttcc
PspCas13b	FFLuc guide-3	caccggcaagaatagctcctcctcgaagcggtaca	caactgtaccgcttcgaggaggagctattcttgcc
PspCas13b	FFLuc guide-4	caccgaagggcaccaccttgcctactgcgccaggc	caacgcctggcgcagtaggcaaggtggtgcccttc
PspCas13b	RLuc guide-1	caccgtgccggacttacccattccgatcagatcag	caacctgatctgatcggaatgggtaagtccggcac
PspCas13b	RLuc guide-2	caccgactcgtcccaggactcgatcacgtccacga	caactcgtggacgtgatcgagtcctgggacgagtc

Supplementary Table S3
Northern blot probes

Target transcript	Probe sequence	Figure panels
eGFP	GTCACGAACTCCAGCAGGAC	1B, 1C, 2C, 4B, 4C, S3B, S4A, S4B, S4D, S6B, S8A, S8B, S8D
mCherry	CGCCGGTGGAGTGGCGGCCC	1B, 1C, 2C, 3C, 4B, 4C, S3B, S4A, S4B, S4D, S6B, S8A, S8B, S8D, S10B, S10D, S10F
RxCas13d	CTCATTCCTATCAAAGAGGGCCTCAATA	1B, 1C, 3C, S3B
PspCas13b or LwaCas13a (Complementary to HA tag)	TCAGGCACGTCGTAGGGGTA	4B, 4C, S6B
RpL32	GACGCACTCTGTTGTCGATACC	1B, 1C, 2C, 3C, 4B, 4C, 5B, S3B, S4A, S4B, S4C, S4D, S6B, S8A, S8B, S8C, S8D
RxCas13d guide RNA	CCCCGACCAGTTGGTAGGG	S1B
PspCas13b guide RNA	CCTCAAAACTGGACCTTCCACAAC	S1B
LwaCas13a guide RNA	CCCCTTCGTTTTTGGGGTAGTCTAA	S1B
tRNA:val4:70BCb	GTTTCCGCCCGGGATCGAAC	S1B
Laccase2 minigene (Exon 2)	GTGTAAGATCTGGTTGATTTTGGTACCTTT	5B
nLuc & FFLuc (Complementary to 3x FLAG)	CGATGTCATGATCTTTATAATCACCGTCATGG	S4C, S8C, S9D
nLuc	CGTAACCCCGTCGATTACCA	6B, 6E, 7B, 7D, S9B, S9D, S11B, S11D
FFLuc	GCCCTTCTTGGCCTTAATGAGAATCT	6B, S8B, S9B, S9D
RLuc	GCGTCCTCCTGGCTGAAGT	7B, 7D, S10B, S10D, S10F, S11B, S11D
RxCas13d & PspCas13b-2A-eGFP (Complementary to HA tag)	AGCGTAATCTGGAACATCGTATGGGTA	6B, 6E, 7B, 7D, S9B, S10B, S10D, S10F, S11B, S11D
GAPDH	GTTGCTGTAGCCAAATTCGTTGT	6B, 6E, 7B, 7D, S9B, S9D, S10B, S10D, S10F, S11B, S11D

Supplementary Table S4
RT-qPCR primers

Target RNA	Forward primer	Reverse primer
eGFP	CCTGAAGTTCATCTGCACCA	AAGTCGTGCTGCTTCATGTG
mCherry	CCTGTCCCCTCAGTTCATGT	CCGTCCTCGAAGTTCATCAC
Drosophila Act42a	CTCCGTCCACCATGAAGATT	TTCGAGATCCACATCTGCTG
FFLuc	GCAGTACCGGATTGCCCAAG	GTCGGGGATGATCTGGTTGC
Human GAPDH	GGTGGTCTCCTCTGACTTCAACA	GTTGCTGTAGCCAAATTCGTTGT

Supplementary Table S5

Full statistical analyses for titration assays

ns (not significant), * p<0.05; ** p<0.01, *** p<0.001, **** p<0.0001

Figure 3C: eGFP mRNA (eGFP guide-2)

Tukey's multiple comparisons test	Mean Diff.	95.00% CI of diff.	Summary	Adjusted P Value
50 ng vs. 25 ng	-0.008231	-0.05932 to 0.04286	ns	0.982
50 ng vs. 10 ng	-0.03191	-0.08300 to 0.01918	ns	0.3086
50 ng vs. 5 ng	-0.02127	-0.07236 to 0.02982	ns	0.6578
50 ng vs. 2 ng	-0.06465	-0.1157 to -0.01356	*	0.013
25 ng vs. 10 ng	-0.02368	-0.07477 to 0.02741	ns	0.5703
25 ng vs. 5 ng	-0.01304	-0.06413 to 0.03805	ns	0.9118
25 ng vs. 2 ng	-0.05642	-0.1075 to -0.005328	*	0.0294
10 ng vs. 5 ng	0.01064	-0.04045 to 0.06173	ns	0.9553
10 ng vs. 2 ng	-0.03274	-0.08383 to 0.01835	ns	0.2877
5 ng vs. 2 ng	-0.04337	-0.09447 to 0.007715	ns	0.1074

Figure 3C: eGFP mRNA (eGFP guide-3)

Tukey's multiple comparisons test	Mean Diff.	95.00% CI of diff.	Summary	Adjusted P Value
50 ng vs. 25 ng	0.02554	-0.04430 to 0.09537	ns	0.7499
50 ng vs. 10 ng	-0.03679	-0.1066 to 0.03305	ns	0.4574
50 ng vs. 5 ng	-0.03601	-0.1058 to 0.03383	ns	0.4767
50 ng vs. 2 ng	-0.06428	-0.1341 to 0.005560	ns	0.075
25 ng vs. 10 ng	-0.06233	-0.1322 to 0.007509	ns	0.0864
25 ng vs. 5 ng	-0.06154	-0.1314 to 0.008291	ns	0.0914
25 ng vs. 2 ng	-0.08981	-0.1596 to -0.01998	*	0.0117
10 ng vs. 5 ng	0.000782	-0.06905 to 0.07062	ns	>0.9999
10 ng vs. 2 ng	-0.02749	-0.09732 to 0.04235	ns	0.6999
5 ng vs. 2 ng	-0.02827	-0.09810 to 0.04157	ns	0.6793

Figure 3D: mCherry mRNA (Random guide)

Tukey's multiple comparisons test	Mean Diff.	95.00% CI of diff.	Summary	Adjusted P Value
Empty vector vs. 0 ng	0.1339	-0.1723 to 0.4400	ns	0.7446
Empty vector vs. 2 ng	0.1275	-0.1786 to 0.4337	ns	0.782
Empty vector vs. 5 ng	0.113	-0.1932 to 0.4191	ns	0.8587
Empty vector vs. 10 ng	0.2092	-0.09699 to 0.5153	ns	0.2944
Empty vector vs. 25 ng	0.1615	-0.1447 to 0.4676	ns	0.5678
Empty vector vs. 50 ng	0.2256	-0.08056 to 0.5318	ns	0.225
0 ng vs. 2 ng	-0.006333	-0.3125 to 0.2998	ns	>0.9999
0 ng vs. 5 ng	-0.0209	-0.3271 to 0.2853	ns	>0.9999
0 ng vs. 10 ng	0.0753	-0.2309 to 0.3815	ns	0.976
0 ng vs. 25 ng	0.0276	-0.2786 to 0.3338	ns	>0.9999
0 ng vs. 50 ng	0.09173	-0.2144 to 0.3979	ns	0.94
2 ng vs. 5 ng	-0.01457	-0.3207 to 0.2916	ns	>0.9999
2 ng vs. 10 ng	0.08163	-0.2245 to 0.3878	ns	0.9647
2 ng vs. 25 ng	0.03393	-0.2722 to 0.3401	ns	0.9997

2 ng vs. 50 ng	0.09807	-0.2081 to 0.4042	ns	0.9199
5 ng vs. 10 ng	0.0962	-0.2100 to 0.4024	ns	0.9262
5 ng vs. 25 ng	0.0485	-0.2577 to 0.3547	ns	0.9976
5 ng vs. 50 ng	0.1126	-0.1935 to 0.4188	ns	0.8603
10 ng vs. 25 ng	-0.0477	-0.3539 to 0.2585	ns	0.9978
10 ng vs. 50 ng	0.01643	-0.2897 to 0.3226	ns	>0.9999
25 ng vs. 50 ng	0.06413	-0.2420 to 0.3703	ns	0.9893

Figure 3D: RxCas13d mRNA (Random guide)

Tukey's multiple comparisons test	Mean Diff.	95.00% CI of diff.	Summary	Adjusted P Value
0 ng vs. 2 ng	-0.03487	-0.3933 to 0.3235	ns	0.9994
0 ng vs. 5 ng	0.06207	-0.2963 to 0.4205	ns	0.9904
0 ng vs. 10 ng	0.01463	-0.3438 to 0.3730	ns	>0.9999
0 ng vs. 25 ng	0.05043	-0.3080 to 0.4088	ns	0.9963
0 ng vs. 50 ng	-0.207	-0.5654 to 0.1514	ns	0.4257
2 ng vs. 5 ng	0.09693	-0.2615 to 0.4553	ns	0.937
2 ng vs. 10 ng	0.0495	-0.3089 to 0.4079	ns	0.9966
2 ng vs. 25 ng	0.0853	-0.2731 to 0.4437	ns	0.9622
2 ng vs. 50 ng	-0.1721	-0.5305 to 0.1863	ns	0.606
5 ng vs. 10 ng	-0.04743	-0.4058 to 0.3110	ns	0.9972
5 ng vs. 25 ng	-0.01163	-0.3700 to 0.3468	ns	>0.9999
5 ng vs. 50 ng	-0.269	-0.6274 to 0.08936	ns	0.1922
10 ng vs. 25 ng	0.0358	-0.3226 to 0.3942	ns	0.9993
10 ng vs. 50 ng	-0.2216	-0.5800 to 0.1368	ns	0.3588
25 ng vs. 50 ng	-0.2574	-0.6158 to 0.1010	ns	0.2259

Figure 3D: mCherry mRNA (eGFP guide-2)

Tukey's multiple comparisons test	Mean Diff.	95.00% CI of diff.	Summary	Adjusted P Value
random vs. 0 ng	-0.0484	-0.2995 to 0.2027	ns	0.9968
random vs. 2 ng	0.1311	-0.1200 to 0.3822	ns	0.624
random vs. 5 ng	0.2857	0.03463 to 0.5368	*	0.0201
random vs. 10 ng	0.4173	0.1662 to 0.6684	***	0.0006
random vs. 25 ng	0.547	0.2959 to 0.7981	****	<0.0001
random vs. 50 ng	0.5841	0.3330 to 0.8352	****	<0.0001
0 ng vs. 2 ng	0.1795	-0.07157 to 0.4306	ns	0.2722
0 ng vs. 5 ng	0.3341	0.08303 to 0.5852	**	0.0055
0 ng vs. 10 ng	0.4657	0.2146 to 0.7168	***	0.0002
0 ng vs. 25 ng	0.5954	0.3443 to 0.8465	****	<0.0001
0 ng vs. 50 ng	0.6325	0.3814 to 0.8836	****	<0.0001
2 ng vs. 5 ng	0.1546	-0.09650 to 0.4057	ns	0.4367
2 ng vs. 10 ng	0.2862	0.03510 to 0.5373	*	0.0199
2 ng vs. 25 ng	0.4159	0.1648 to 0.6670	***	0.0006
2 ng vs. 50 ng	0.4529	0.2018 to 0.7040	***	0.0002
5 ng vs. 10 ng	0.1316	-0.1195 to 0.3827	ns	0.6202
5 ng vs. 25 ng	0.2613	0.01016 to 0.5124	*	0.0384
5 ng vs. 50 ng	0.2983	0.04723 to 0.5494	*	0.0144
10 ng vs. 25 ng	0.1297	-0.1214 to 0.3808	ns	0.636
10 ng vs. 50 ng	0.1667	-0.08437 to 0.4178	ns	0.3507
25 ng vs. 50 ng	0.03707	-0.2140 to 0.2882	ns	0.9994

Figure 3D: RxCas13d mRNA (eGFP guide-2)

Tukey's multiple comparisons test	Mean Diff.	95.00% CI of diff.	Summary	Adjusted P Value
random vs. 0 ng	-0.1144	-0.5221 to 0.2932	ns	0.9552
random vs. 2 ng	-0.1176	-0.5253 to 0.2901	ns	0.9493
random vs. 5 ng	0.3192	-0.08846 to 0.7269	ns	0.1762
random vs. 10 ng	0.4062	-0.001491 to 0.8138	ns	0.0511
random vs. 25 ng	0.6398	0.2321 to 1.047	**	0.0015
random vs. 50 ng	0.6586	0.2510 to 1.066	**	0.0011
0 ng vs. 2 ng	-0.003167	-0.4108 to 0.4045	ns	>0.9999
0 ng vs. 5 ng	0.4336	0.02598 to 0.8413	*	0.0338
0 ng vs. 10 ng	0.5206	0.1129 to 0.9283	**	0.009
0 ng vs. 25 ng	0.7542	0.3465 to 1.162	***	0.0003
0 ng vs. 50 ng	0.7731	0.3654 to 1.181	***	0.0002
2 ng vs. 5 ng	0.4368	0.02914 to 0.8445	*	0.0323
2 ng vs. 10 ng	0.5238	0.1161 to 0.9314	**	0.0086
2 ng vs. 25 ng	0.7574	0.3497 to 1.165	***	0.0003
2 ng vs. 50 ng	0.7762	0.3686 to 1.184	***	0.0002
5 ng vs. 10 ng	0.08697	-0.3207 to 0.4946	ns	0.9882
5 ng vs. 25 ng	0.3206	-0.08709 to 0.7282	ns	0.173
5 ng vs. 50 ng	0.3394	-0.06822 to 0.7471	ns	0.1339
10 ng vs. 25 ng	0.2336	-0.1741 to 0.6413	ns	0.479
10 ng vs. 50 ng	0.2525	-0.1552 to 0.6601	ns	0.3952
25 ng vs. 50 ng	0.01887	-0.3888 to 0.4265	ns	>0.9999

Figure 3D: mCherry mRNA (eGFP guide-3)

Tukey's multiple comparisons test	Mean Diff.	95.00% CI of diff.	Summary	Adjusted P Value
random vs. 0 ng	0.07227	-0.1940 to 0.3385	ns	0.9769
random vs. 2 ng	0.2298	-0.03641 to 0.4961	ns	0.118
random vs. 5 ng	0.2594	-0.006840 to 0.5256	ns	0.059
random vs. 10 ng	0.3708	0.1045 to 0.6370	**	0.0036
random vs. 25 ng	0.5342	0.2680 to 0.8004	****	<0.0001
random vs. 50 ng	0.655	0.3887 to 0.9212	****	<0.0001
0 ng vs. 2 ng	0.1576	-0.1087 to 0.4238	ns	0.4826
0 ng vs. 5 ng	0.1871	-0.07911 to 0.4534	ns	0.2896
0 ng vs. 10 ng	0.2985	0.03226 to 0.5647	*	0.0225
0 ng vs. 25 ng	0.4619	0.1957 to 0.7282	***	0.0004
0 ng vs. 50 ng	0.5827	0.3165 to 0.8489	****	<0.0001
2 ng vs. 5 ng	0.02957	-0.2367 to 0.2958	ns	>0.9999
2 ng vs. 10 ng	0.1409	-0.1253 to 0.4072	ns	0.6093
2 ng vs. 25 ng	0.3044	0.03813 to 0.5706	*	0.0194
2 ng vs. 50 ng	0.4251	0.1589 to 0.6914	***	0.0009
5 ng vs. 10 ng	0.1114	-0.1549 to 0.3776	ns	0.8224
5 ng vs. 25 ng	0.2748	0.008560 to 0.5410	*	0.0405
5 ng vs. 50 ng	0.3956	0.1293 to 0.6618	**	0.0019
10 ng vs. 25 ng	0.1634	-0.1028 to 0.4297	ns	0.4401
10 ng vs. 50 ng	0.2842	0.01796 to 0.5504	*	0.0321
25 ng vs. 50 ng	0.1208	-0.1455 to 0.3870	ns	0.7602

Figure 3D: RxCas13d mRNA (eGFP guide-3)

Tukey's multiple comparisons test	Mean Diff.	95.00% CI of diff.	Summary	Adjusted P Value

random vs. 0 ng	0.2312	-0.1010 to 0.5634	ns	0.2765
random vs. 2 ng	0.4231	0.09092 to 0.7553	**	0.0092
random vs. 5 ng	0.3963	0.06406 to 0.7285	*	0.0152
random vs. 10 ng	0.4941	0.1619 to 0.8263	**	0.0025
random vs. 25 ng	0.59	0.2578 to 0.9222	***	0.0005
random vs. 50 ng	0.6841	0.3519 to 1.016	****	<0.0001
0 ng vs. 2 ng	0.1919	-0.1403 to 0.5241	ns	0.4703
0 ng vs. 5 ng	0.165	-0.1672 to 0.4972	ns	0.6289
0 ng vs. 10 ng	0.2629	-0.06934 to 0.5951	ns	0.1684
0 ng vs. 25 ng	0.3587	0.02652 to 0.6909	*	0.0306
0 ng vs. 50 ng	0.4528	0.1206 to 0.7850	**	0.0053
2 ng vs. 5 ng	-0.02687	-0.3591 to 0.3053	ns	>0.9999
2 ng vs. 10 ng	0.07097	-0.2612 to 0.4032	ns	0.9881
2 ng vs. 25 ng	0.1668	-0.1654 to 0.4990	ns	0.6181
2 ng vs. 50 ng	0.2609	-0.07128 to 0.5931	ns	0.1738
5 ng vs. 10 ng	0.09783	-0.2344 to 0.4300	ns	0.9444
5 ng vs. 25 ng	0.1937	-0.1385 to 0.5259	ns	0.4602
5 ng vs. 50 ng	0.2878	-0.04441 to 0.6200	ns	0.1105
10 ng vs. 25 ng	0.09587	-0.2363 to 0.4281	ns	0.9492
10 ng vs. 50 ng	0.19	-0.1422 to 0.5222	ns	0.4813
25 ng vs. 50 ng	0.0941	-0.2381 to 0.4263	ns	0.9533

Figure 6D: FFLuc mRNA (FFLuc guide-1)

Tukey's multiple comparisons test	Mean Diff.	95.00% CI of diff.	Summary	Adjusted P Value
150 ng vs. 50 ng	-0.05945	-0.3725 to 0.2536	ns	0.9267
150 ng vs. 20 ng	-0.2568	-0.5698 to 0.05633	ns	0.1127
50 ng vs. 20 ng	-0.1973	-0.5104 to 0.1158	ns	0.2579

Figure 6D: FFLuc mRNA (FFLuc guide-4)

Tukey's multiple comparisons test	Mean Diff.	95.00% CI of diff.	Summary	Adjusted P Value
150 ng vs. 50 ng	-0.1122	-0.4961 to 0.2717	ns	0.7874
150 ng vs. 20 ng	-0.2991	-0.6830 to 0.08484	ns	0.1354
50 ng vs. 20 ng	-0.1869	-0.5708 to 0.1970	ns	0.4502

Figure 6E: nLuc mRNA (Random guide)

Tukey's multiple comparisons test	Mean Diff.	95.00% CI of diff.	Summary	Adjusted P Value
0 ng vs. 20 ng	-0.08253	-0.4622 to 0.2971	ns	0.9998
0 ng vs. 50 ng	-0.0099	-0.3895 to 0.3697	ns	>0.9999
0 ng vs. 150 ng	-0.04517	-0.4248 to 0.3345	ns	>0.9999
20 ng vs. 50 ng	0.07263	-0.3070 to 0.4523	ns	>0.9999
20 ng vs. 150 ng	0.03737	-0.3423 to 0.4170	ns	>0.9999
50 ng vs. 150 ng	-0.03527	-0.4149 to 0.3444	ns	>0.9999

Figure 6E: RxCas13d mRNA (Random guide)

Tukey's multiple comparisons test	Mean Diff.	95.00% CI of diff.	Summary	Adjusted P Value
0 ng vs. 20 ng	-0.1916	-0.5599 to 0.1767	ns	0.7624
0 ng vs. 50 ng	0.07003	-0.2983 to 0.4383	ns	0.9999
0 ng vs. 150 ng	-0.0031	-0.3714 to 0.3652	ns	>0.9999
20 ng vs. 50 ng	0.2616	-0.1067 to 0.6299	ns	0.3522

20 ng vs. 150 ng	0.1885	-0.1798 to 0.5568	ns	0.7791
50 ng vs. 150 ng	-0.07313	-0.4414 to 0.2952	ns	0.9998

Figure 6E: nLuc mRNA (FFLuc guide-1)

Tukey's multiple comparisons test	Mean Diff.	95.00% CI of diff.	Summary	Adjusted P Value
0 ng vs. 20 ng	0.2029	-0.07829 to 0.4841	ns	0.1994
0 ng vs. 50 ng	0.4755	0.1943 to 0.7567	**	0.0017
0 ng vs. 150 ng	0.6615	0.3803 to 0.9427	***	0.0001
20 ng vs. 50 ng	0.2726	-0.008560 to 0.5538	ns	0.0584
20 ng vs. 150 ng	0.4586	0.1774 to 0.7398	**	0.0023
50 ng vs. 150 ng	0.1859	-0.09526 to 0.4671	ns	0.2629

Figure 6E: RxCas13d mRNA (FFLuc guide-1)

Tukey's multiple comparisons test	Mean Diff.	95.00% CI of diff.	Summary	Adjusted P Value
0 ng vs. 20 ng	0.143	-0.1487 to 0.4346	ns	0.5217
0 ng vs. 50 ng	0.5536	0.2619 to 0.8453	***	0.0007
0 ng vs. 150 ng	0.7727	0.4810 to 1.064	****	<0.0001
20 ng vs. 50 ng	0.4106	0.1190 to 0.7023	**	0.0065
20 ng vs. 150 ng	0.6297	0.3381 to 0.9214	***	0.0002
50 ng vs. 150 ng	0.2191	-0.07258 to 0.5108	ns	0.1733

Figure 6E: nLuc mRNA (FFLuc guide-4)

Tukey's multiple comparisons test	Mean Diff.	95.00% CI of diff.	Summary	Adjusted P Value
0 ng vs. 20 ng	0.09387	-0.2573 to 0.4450	ns	0.898
0 ng vs. 50 ng	0.3258	-0.02533 to 0.6769	ns	0.0722
0 ng vs. 150 ng	0.5286	0.1775 to 0.8798	**	0.004
20 ng vs. 50 ng	0.2319	-0.1192 to 0.5831	ns	0.2637
20 ng vs. 150 ng	0.4348	0.08363 to 0.7859	*	0.0149
50 ng vs. 150 ng	0.2028	-0.1483 to 0.5540	ns	0.3754

Figure 6E: RxCas13d mRNA (FFLuc guide-4)

Tukey's multiple comparisons test	Mean Diff.	95.00% CI of diff.	Summary	Adjusted P Value
0 ng vs. 20 ng	0.3321	0.09078 to 0.5734	**	0.0075
0 ng vs. 50 ng	0.6583	0.4170 to 0.8996	****	<0.0001
0 ng vs. 150 ng	0.8895	0.6482 to 1.131	****	<0.0001
20 ng vs. 50 ng	0.3263	0.08498 to 0.5676	**	0.0085
20 ng vs. 150 ng	0.5575	0.3162 to 0.7988	***	0.0001
50 ng vs. 150 ng	0.2312	-0.01009 to 0.4725	ns	0.0619

SUPPLEMENTARY METHODS

The following *Drosophila* expression plasmids were each generated from the previously published **pUb 3xFLAG MCS** plasmid (Chen et al. (2012) *RNA*, **18**, 2148-2156.) using the three steps outlined below.

pUb RxCas13d + RxCas13d guide RNA (Addgene #176303)

pUb dRxCas13d + RxCas13d guide RNA (Addgene #176304)

pUb PspCas13b + PspCas13b guide RNA (Addgene #176305)

pUb dPspCas13b + PspCas13b guide RNA (Addgene #176306)

pUb LwaCas13a + LwaCas13a guide RNA (Addgene #176307)

The full plasmid sequence of **pUb 3xFLAG MCS** is as follows:

GACGAAAGGGCCTCGTGATACGCCTATTTTTATAGGTTAATGTCATGATAATAATGGTTTCTTAGACGTCAGGTGGCACTTTTCGGGGAAATGTGCG
CGGAACCCCTATTTGTTTATTTTTCTAAATACATTCAAATATGTATCCGCTCATGAGACAATAACCCTGATAAATGCTTCAATAATATTGAAAAAGG
AAGAGTATGAGTATTCAACATTTCCGTGTCGCCCTTATTCCCTTTTTTGCGGCATTTTGCCTTCCTGTTTTTGCTCACCCAGAAACGCTGGTGAAAG
TAAAAGATGCTGAAGATCAGTTGGGTGCACGAGTGGGTTACATCGAACTGGATCTCAACAGCGGTAAGATCCTTGAGAGTTTTCGCCCCGAAGAACG
TTTTCCAATGATGAGCACTTTTAAAGTTCTGCTATGTGGCGCGGTATTATCCCGTATTGACGCCGGGCAAGAGCAACTCGGTCGCCGCATACACTAT
TCTCAGAATGACTTGGTTGAGTACTCACCAGTCACAGAAAAGCATCTTACGGATGGCATGACAGTAAGAGAATTATGCAGTGCTGCCATAACCATGA
GTGATAACACTGCGGCCAACTTACTTCTGACAACGATCGGAGGACCGAAGGAGCTAACCGCTTTTTTGCACAACATGGGGGATCATGTAACTCGCCT
TGATCGTTGGGAACCGGAGCTGAATGAAGCCATACCAAACGACGAGCGTGACACCACGATGCCTGTAGCAATGGCAACAACGTTGCGCAAACTATTA
ACTGGCGAACTACTTACTCTAGCTTCCCGGCAACAATTAATAGACTGGATGGAGGCGGATAAAGTTGCAGGACCACTTCTGCGCTCGGCCCTTCCGG
CTGGCTGGTTTATTGCTGATAAATCTGGAGCCGGTGAGCGTGGGTCTCGCGGTATCATTGCAGCACTGGGGCCAGATGGTAAGCCCTCCCGTATCGT
AGTTATCTACACGACGGGGAGTCAGGCAACTATGGATGAACGAAATAGACAGATCGCTGAGATAGGTGCCTCACTGATTAAGCATTGGTAACTGTCA
GACCAAGTTTACTCATATATACTTTAGATTGATTTAAAACTTCATTTTTAATTTAAAAGGATCTAGGTGAAGATCCTTTTTGATAATCTCATGACCA
AAATCCCTTAACGTGAGTTTTCGTTCCACTGAGCGTCAGACCCCGTAGAAAAGATCAAAGGATCTTCTTGAGATCCTTTTTTTCTGCGCGTAATCTG
CTGCTTGCAAACAAAAAAACCACCGCTACCAGCGGTGGTTTGTTTGCCGGATCAAGAGCTACCAACTCTTTTTCCGAAGGTAACTGGCTTCAGCAGA
GCGCAGATACCAAATACTGTTCTTCTAGTGTAGCCGTAGTTAGGCCACCACTTCAAGAACTCTGTAGCACCGCCTACATACCTCGCTCTGCTAATCC
TGTTACCAGTGGCTGCTGCCAGTGGCGATAAGTCGTGTCTTACCGGGTTGGACTCAAGACGATAGTTACCGGATAAGGCGCAGCGGTCGGGCTGAAC
GGGGGGTTCGTGCACACAGCCCAGCTTGGAGCGAACGACCTACACCGAACTGAGATACCTACAGCGTGAGCTATGAGAAAGCGCCACGCTTCCCGAA
GGGAGAAAGGCGGACAGGTATCCGGTAAGCGGCAGGGTCGGAACAGGAGAGCGCACGAGGGAGCTTCCAGGGGGAAACGCCTGGTATCTTTATAGTC
CTGTCGGGTTTCGCCACCTCTGACTTGAGCGTCGATTTTTGTGATGCTCGTCAGGGGGGCGGAGCCTATGGAAAAACGCCAGCAACGCGGCCTTTTT
ACGGTTCCTGGCCTTTTGCTGGCCTTTTGCTCACATGTTCTTTCCTGCGTTATCCCCTGATTCTGTGGATAACCGTATTACCGCCTTTGAGTGAGCT
GATACCGCTCGCCGCAGCCGAACGACCGAGCGCAGCGAGTCAGTGAGCGAGGAAGCGGAAGAGCGCCCAATACGCAAACCGCCTCTCCCCGCGCGTT
GGCCGATTCATTAATGCAGCTGGCACGACAGGTTTCCCGACTGGAAAGCGGGCAGTGAGCGCAACGCAATTAATGTGAGTTAGCTCACTCATTAGGC
ACCCCAGGCTTTACACTTTATGCTTCCGGCTCGTATGTTGTGTGGAATTGTGAGCGGATAACAATTTCACACAGGAAACAGCTATGACCATGATTAC
GCCAAAGCTTGTCGCCGGAACGCAGCGACAGAGATTCCAATGTGTCCGTATCTTTCAGGCTTTTGCCCTTCAGTTCCAGACGAAGCGACTGGCGATT
CGCGTGTGGGGTCTGCTTCAGGGTCTTGTGAATTAGGGCGCGCAGATCGCCGATGGGCGTGGCGCCGGAGGGCACCTTCACCTTGCCGTACGGCTTG
CTGTTCTTCGCGTTCAAAATCTCCAGCTCCATTTTGCTTTCGGTGCGCTTGCAATCAGTACTGTCCAAAATCGAAAATCGCCGAACCGTAGTGTGAC
CGTGCGGGGCTCTGCGAAAATAAACTTTTTTAGGTATATGGCCACACACGGGGAAAGCACAGTGGATTATATGTTTTAATATTATAATATGCAGGTT
TTCATTACTTATCCAGATGTAAGCCCACTTAAAGCGATTTAACAATTATTTGCCGAAAGAGTATAAACAAATTTCACTTAAAAATGGATTAAGAAAA
GCTAGCTTGTGTAAGATTATGCGCAGCGTTGCCAGATAGCTCCATTTAAAACACTTCAAAAACAATAAGTTTTGAAAATATATACATAAATAGCAGT
CGTTGCCGCAACGCTCAACACATCACACTTTTAAAACACCCTTTACCTACACAGAATTACTTTTTAAATTTCCAGTCAAGCTGCGAGTTTCAAAATT
ATAGCCGGTAGAGAAGACAGTGCTATTTCAAAAGCAAACTAACAAGGGTCTTAAATTCCAAAACACCAATCCTAACAAGCCTTGGACTTTTGTAAGT
TTAGATCAAAGGTGGCATTGCATTCAATGTCATGGTAAGAAGTAGGTCGTCTAGGTAGAAATCCTCATTCAGCCGGTCAAGTCAGTACGAGAAAGGT
CTCAATTTGAAATTGTCTTAAAAATATTTTATTGTTTTGTACTGTGGTGAGTTTAAACGAAAAACACAAAAAAAAAGTGATACACAGAAATCATAAA
AAATTTTAATACAAGGTATTCGTACGTATCAAAAACATTTCGGCACAATTTTTTTTCTCTGTACTAAAGTGTTACGAACACTACGGTATTTTTTAGT
GATTTTCAACGGACACCGAAGGTATATAAACAGCGTTCGCGAACGGTCGCCTTCAAAACCAATTGACATTTGCAGCAGCAAGTACAAGTAGAAAGTA
AAGCGCAATCAGCGAAAAATTTATACTTAATTGTTGGTGATTAAAGTACAATTAAAAGAACATTCTCGAAAGTCACAAGAAACGTAAGTTTTTAACT
CGCTGTTACCAATTAGTAATAAGAGCAACAAGACGTTGAGTAATTTCAAGAAAAACTGCATTTCAAGGTCTTTGTTCGGCCATTTTTTTTTATTCAA
CGCTCTACGTAATTACAAAATAAGAAATTGGCAGCCACGCATCTTGTTTTCCCAATGAATTGGCATCAAAACGCAAACAAATCTATAAATAAAACTT
GCGTGTTGATTTTCGCCAAGATTTATTGGCAAATTGTGAAATTCGCAGTGACGCATTTGAAAATTCGAGAAATCACGAACGCACTCGATCGAGCATT
TGTGTGCATGTTATTAGTTAGTTGTTTAGTTAATTGAAGTATTTTACCAACGAAATCCACTTATTTTTAGCTGAAATAGAGTAGGTTGCTTAAACAA
AGCCACGTCTGAAAATTTCTTATTGCTTGTAGTTGTGACGTCACCATATACACACAAAATAATGTGTATGCATGCATTTCAGCTGTGTATATATACA

TGCACACACTCGCAACACGAAAACGATGACGAAGCAACGGAACAAAGGTTTCTCAACTACCCTTTGTTCCCTGTTTCTTCGCTTTCCTTTGTTCCAA
TATTCGTAGAGGGTTAATAGGGGTTTCTCAACAAAGTTGGCGTCGATAAATAAGTTTCCCATTTTTATTCCCCAGCCAGGAAGTTAGTTTCAATAGT
TTTGTAATTTCAACGAAACTCATTTGATTTCGTACTAATTTTCCACATCTCTATTTTCTTCCCGCAGAATAATCCAAACTGCAGGTCGACTCTAGCT
AGAGGAAGCTTATGGACTACAAAGACCATGACGGTGATTATAAAGATCATGATATCGATTACAAGGATGACGATGACAAGGATCCACTAGTCCAGTG
TGGTGGAATTCTGCAGATATCCAGCACAGTGGCGGCCGCTCGAGTCTAGAGGGCCCGCGGTTCGAAGGTAAGCCTATCCCTAACCCTCTCCTCGGTC
TCGATTCTACGCGTACCGGTCATCATCACCATCACCATTGAGTTTATCTGACTAAATCTTAGTTTGTATTGTCATGTTTTAATACAATATGTTATGT
TTAAATATGTTTTTAATAAATTTTATAAAATAATTTCAACTTTTATTGTAACAACATTGTCCATTTACACACTCCTTTCAAGCGCGTGGGATCGATG
CTCACTCAAAGGCGGTAATACGGTTATCCACAGAATCAGGGGATAACGCAGGAAAGAACATGTGAGCCATATGGGCCCATGTGAGCCATATGGTGCA
CTCTCAGTACAATCTGCTCTGATGCCGCATAGTTAAGCCAGCCCCGACACCCGCCAACACCCGCTGACGCGCCCTGACGGGCTTGTCTGCTCCCGGC
ATCCGCTTACAGACAAGCTG**TGACCGTCTCCGGGAGCTGCATGTGTCAGAGGTTTTCACCGTCATCACCGAAACGCGCGA**

pUbi-p63e (pUb) promoter

CGTCTC: BsmBI site

CATATG: NdeI site

GAACGTTTTC: XmnI site

Step 1:

The BsmBI restriction site (yellow) and surrounding nucleotides (in bold above) were first removed to generate the **pUb 3xFLAG MCS (No BsmBI)** plasmid. This was done by cutting **pUb 3xFLAG MCS** with NdeI and XmnI and inserting the following sequence between the NdeI and XmnI sites.

GGCCCATGTGAGCCATATGGTGCACTCTCAGTACAATCTGCTCTGATGCCGCATAGTTAAGCCAGCCCCGACACCCGCCAACACCCGCTGACGCGCC
CTGACGGGCTTGTCTGCTCCCGGCATCCGCTTACAGACAAGCTGTGTCATGATAATAATGGTTTCTTAGACGTCAGGTGGCACTTTTCGGGGAAATG
TGCGCGGAACCCCTATTTGTTTATTTTTCTAAATACATTCAAATATGTATCCGCTCATGAGACAATAACCCTGATAAATGCTTCAATAATATTGAAA
AAGGAAGAGTATGAGTATTCAACATTTCCGTGTCGCCCTTATTCCCTTTTTTGCGGCATTTTGCCTTCCTGTTTTTGCTCACCCAGAAACGCTGGTG
AAAGTAAAAGATGCTGAAGATCAGTTGGGTGCACGAGTGGGTTACATCGAACTGGATCTCAACAGCGGTAAGATCCTTGAGAGTTTTCGCCCCGAA

Step 2:

A guide RNA sequence containing BsmBI restriction sites that is driven by the snRNA:U6:96Ab promoter was inserted into the NdeI site.

pUb 3xFLAG MCS (No BsmBI) + RxCas13d guide RNA was made by inserting the following sequence into the NdeI site in **pUb 3xFLAG MCS (No BsmBI)**:

gttcgacttgcagcctgaaatacggcacgagtaggaaaagccgagtcaaatgccgaatgcagagtctcattacagcacaatcaactcaagaaaaact
cgacactttttaccatttgcacttaaatcctttttattcgttatgtatactttttttggtccctaaccaaaacaaaaccaaactctcttagtcgt
gcctctatatttaaaactatcaatttattatagtcaataaatcgaactgtgttttcaacaaacgaacaataggacactttgattctaaaggaaattt
tgaaaatcttaagcagagggttcttaagaccatttgccaattcttataattctcaactgctctttcctgatgttgatcatttatataggtatgtttt
cctcaatacttcgaacccctaccaactggtcggggtttgaaacgagacggttgtatttgtacgtctctttttt

snRNA:U6:96Ab Promoter
Terminator
RxCas13d guide RNA scaffold
BsmBI sites

pUb 3xFLAG MCS (No BsmBI) + PspCas13b guide RNA was made by inserting the following sequence into the NdeI site in **pUb 3xFLAG MCS (No BsmBI)**:

Gttcgacttgcagcctgaaatacggcacgagtaggaaaagccgagtcaaatgccgaatgcagagtctcattacagcacaatcaactcaagaaaaact
cgacactttttaccatttgcacttaaatcctttttattcgttatgtatactttttttggtccctaaccaaaacaaaaccaaactctcttagtcgt
gcctctatatttaaaactatcaatttattatagtcaataaatcgaactgtgttttcaacaaacgaacaataggacactttgattctaaaggaaattt
tgaaaatcttaagcagagggttcttaagaccatttgccaattcttataattctcaactgctctttcctgatgttgatcatttatataggtatgtttt
cctcaatacttcggagacggttgtatttgtagttgtacgtctcAgttgtggaaggtccagttttgaggggctattacaacttttttt

snRNA:U6:96Ab Promoter
Terminator
PspCas13b guide RNA scaffold
BsmBI sites

pUb 3xFLAG MCS (No BsmBI) + LwaCas13a guide RNA was made by inserting the following sequence into the NdeI site in **pUb 3xFLAG MCS (No BsmBI)**:

Gttcgacttgcagcctgaaatacggcacgagtaggaaaagccgagtcaaatgccgaatgcagagtctcattacagcacaatcaactcaagaaaaact
cgacactttttaccatttgcacttaaatcctttttattcgttatgtatacttttttggtccctaaccaaaacaaaaccaaactctcttagtcgt
gcctctatatttaaaactatcaatttattatagtcaataaatcgaactgtgttttcaacaaacgaacaataggacactttgattctaaaggaaattt
tgaaaatcttaagcagagggttcttaagaccatttgccaattcttataattctcaactgctctttcctgatgttgatcatttatataggtatgtttt
cctcaatacttcgGATTTAGACTACCCCAAAAACGAAGGGGACTAAAACgagacggttgtatttgtagttgcgtctcTTTTTTT

snRNA:U6:96Ab Promoter
Terminator
LwaCas13a guide RNA scaffold
BsmBI sites

Step 3:

The indicated Cas13 ORF was inserted downstream of the Ubi-p63e promoter.

pUb RxCas13d + RxCas13d guide RNA was made by inserting the RxCas13d ORF between SalI and AgeI in **pUb 3xFLAG MCS (No BsmBI) + RxCas13d guide RNA**:

aagaccatgattgagaagaagaaatcctttgccaaagggatgggcgtgaagagcactctggtgagcggcagcaaggtgtacatgaccacctttgcag
aggggagtgatgcccggctggagaagatcgtggaaggggacagcatccgctctgtgaatgaaggagaagctttcagtgcagaaatggctgacaagaa
tgctgggtacaaaatcgggaacgccaagttcagccaccccaagggatatgcagtggttgctaacaatcctctctacacaggacctgtgcagcaggac
atgctggggctgaaggagactttggagaagcggtatttcggggagtctgcagatggaaatgacaatatctgcattcaggtgattcataatattcttg
atattgaaaagatcctggctgagtacatcacaaatgctgcatatgctgttaataacatctcaggcttggacaaggatattattgggtttgggaagtt
ttccacagtgtatacctacgatgaatttaaagaccccgagcaccacagagctgccttcaacaataatgacaaattgatcaatgccatcaaggctcag
tatgatgaatttgacaacttcctggacaacccaaggctgggctattttggccaggccttcttcagcaaagagggccgcaactacatcattaattacg
ggaatgaatgttatgatatcctcgccctgctctctggcctgaggcactgggtggtccacaacaacgaggaagaaagcaggatttctaggacctggct
gtacaatcttgacaagaatctggacaatgaatatatctccaccctcaactatttgtatgaccggatcaccaacgaactcacaaacagcttttctaag
aactcagccgcaaatgtgaactacattgcagagacactgggcatcaaccccgccgagtttgctgaacagtacttccggttctccattatgaaggagc
agaagaatttgggttttaacattactaaacttagggaggtcatgctcgacaggaaagatatgtctgagatccggaagaaccacaaggtcttcgattc
catcaggacaaaagtctacaccatgatggactttgttatttaccgctattacattgaagaagatgctaaagtggccgccgctaataagagcctgcca
gataatgagaaatctctcagcgagaaggacatctttgtgatcaacctgcggggctccttcaatgatgatcagaaggatgcactgtactacgatgaag
ccaaccgcatctggagaaagctggagaacatcatgcacaatataaaggaattccgcggcaataagacacgggagtacaagaagaaagacgccccacg
cctgcccagaattctgcctgctggccgggatgtctctgccttttcaaaactcatgtatgccttgacaatgtttctggatggcaaagaaatcaatgat
ttgctgacaaccctgatcaacaaatttgacaacatccagagcttcctcaaggtgatgcccctgattggagtaaatgcaaaatttgtcgaggagtatg
ccttcttcaaggactctgccaagattgccgatgagctgcgcctcattaagtcattcgcccgcatgggagagcccatcgcagacgcaagaagagccat
gtacatcgatgccattcgcattttgggcaccaatctctcctatgacgagctcaaagctctggccgacaccttctctttggatgaaaatggcaacaag
cttaagaaggggaaacatggcatgagaaatttcatcatcaacaacgtcatttccaacaagcgcttccactatctcatcagatatggagaccctgccc
acctgcatgaaattgctaagaatgaggctgtggtgaaattcgtgctgggaaggatcgccgacattcaaaagaagcaggggcagaatggaaagaacca
gatcgaccgctactatgaaacctgtatcggaaaagataagggcaagtctgtcagtgagaaagtggacgccctcaccaagatcatcactggaatgaac
tatgaccagttcgacaagaaaagaagtgtgatcgaggacacaggccgggagaatgccgagagagagaagttcaagaaaattatcagcctctatctga
ccgtcatctaccacatcctgaagaacattgtcaacattaatgccaggtacgtgattggctttcactgcgttgaaagagatgcccagctttataagga
gaaaggctacgacattaacttgaagaaactggaggagaaaggatttagcagcgtgaccaaactgtgtgccggcattgacgagaccgcccctgataaa
cggaaagatgtggagaaagagatggccgagcgggccaaggagagcatcgattctctggaaagtgccaaccctaagctctacgccaattatattaaat
attctgatgagaagaaagcagaagagttcacccggcagattaacagggaaaaggccaaaacagccctgaacgcctacctgagaaacaccaagtggaa
tgtcatcattagagaagacctgcttcggattgataacaagacctgcaccctcttcaggaacaaagccgtgcacttggaagtggctcgctatgtgcat
gcctatatcaatgacatcgctgaggtgaactcctacttccagctgtatcactacatcatgcagagaatcattatgaatgaaaggtatgagaaatcat
ctggtaaagtatctgaatactttgatgctgtcaatgatgagaagaagtacaacgacagactcctgaagctgctgtgcgtgccctttggatactgcat
ccccaggtttaagaacctcagtattgaggccctctttgataggaatgaggcagcaaagtttgataaagaaaagaagaaagtttctggaaacagtgga
agtggcgcagctgcttacccctacgacgtgcctgactacgcctga

pUb dRxCas13d + RxCas13d guide RNA was made by inserting the dRxCas13d ORF (catalytic dead mutations are noted in red) between SalI and AgeI in **pUb 3xFLAG MCS (No BsmBI) + RxCas13d guide RNA**:

aagaccatgattgagaagaagaaatcctttgccaaagggatgggcgtgaagagcactctggtgagcggcagcaaggtgtacatgaccacctttgcag
aggggagtgatgcccggctggagaagatcgtggaaggggacagcatccgctctgtgaatgaaggagaagctttcagtgcagaaatggctgacaagaa
tgctgggtacaaaatcgggaacgccaagttcagccaccccaagggatatgcagtggttgctaacaatcctctctacacaggacctgtgcagcaggac
atgctggggctgaaggagactttggagaagcggtatttcggggagtctgcagatggaaatgacaatatctgcattcaggtgattcataatattcttg
atattgaaaagatcctggctgagtacatcacaaatgctgcatatgctgttaataacatctcaggcttggacaaggatattattgggtttgggaagtt
ttccacagtgtatacctacgatgaatttaaagaccccgagcaccacagagctgccttcaacaataatgacaaattgatcaatgccatcaaggctcag
tatgatgaatttgacaacttcctggacaacccaaggctgggctattttggccaggccttcttcagcaaagagggccgcaactacatcattaattacg
ggaatgaatgttatgatatcctcgccctgctctctggcctg**gcc**cactgggtggtc**gcc**aacaacgaggaagaaagcaggatttctaggacctggct
gtacaatcttgacaagaatctggacaatgaatatatctccaccctcaactatttgtatgaccggatcaccaacgaactcacaaacagcttttctaag
aactcagccgcaaatgtgaactacattgcagagacactgggcatcaaccccgccgagtttgctgaacagtacttccggttctccattatgaaggagc
agaagaatttgggttttaacattactaaacttagggaggtcatgctcgacaggaaagatatgtctgagatccggaagaaccacaaggtcttcgattc
catcaggacaaaagtctacaccatgatggactttgttatttaccgctattacattgaagaagatgctaaagtggccgccgctaataagagcctgcca
gataatgagaaatctctcagcgagaaggacatctttgtgatcaacctgcggggctccttcaatgatgatcagaaggatgcactgtactacgatgaag
ccaaccgcatctggagaaagctggagaacatcatgcacaatataaaggaattccgcggcaataagacacgggagtacaagaagaaagacgccccacg
cctgcccagaattctgcctgctggccgggatgtctctgccttttcaaaactcatgtatgccttgacaatgtttctggatggcaaagaaatcaatgat
ttgctgacaaccctgatcaacaaatttgacaacatccagagcttcctcaaggtgatgcccctgattggagtaaatgcaaaatttgtcgaggagtatg
ccttcttcaaggactctgccaagattgccgatgagctgcgcctcattaagtcattcgcccgcatgggagagcccatcgcagacgcaagaagagccat
gtacatcgatgccattcgcattttgggcaccaatctctcctatgacgagctcaaagctctggccgacaccttctctttggatgaaaatggcaacaag
cttaagaaggggaaacatggcatgagaaatttcatcatcaacaacgtcatttccaacaagcgcttccactatctcatcagatatggagaccctgccc
acctgcatgaaattgctaagaatgaggctgtggtgaaattcgtgctgggaaggatcgccgacattcaaaagaagcaggggcagaatggaaagaacca
gatcgaccgctactatgaaacctgtatcggaaaagataagggcaagtctgtcagtgagaaagtggacgccctcaccaagatcatcactggaatgaac
tatgaccagttcgacaagaaaagaagtgtgatcgaggacacaggccgggagaatgccgagagagagagaagttcaagaaaattatcagcctctatctga
ccgtcatctaccacatcctgaagaacattgtcaacattaatgccaggtacgtgattggctttcactgcgttgaaagagatgcccagctttataagga
gaaaggctacgacattaacttgaagaaactggaggagaaaggatttagcagcgtgaccaaactgtgtgccggcattgacgagaccgcccctgataaa
cggaaagatgtggagaaagagatggccgagcgggccaaggagagcatcgattctctggaaagtgccaaccctaagctctacgccaattatattaaat
attctgatgagaagaaagcagaagagttcacccggcagattaacagggaaaaggccaaaacagccctgaacgcctacctgagaaacaccaagtggaa
tgtcatcattagagaagacctgcttcggattgataacaagacctgcaccctcttc**gcc**aacaaagccgt**ggc**cttggaagtggctcgctatgtgcat
gcctatatcaatgacatcgctgaggtgaactcctacttccagctgtatcactacatcatgcagagaatcattatgaatgaaaggtatgagaaatcat
ctggtaaagtatctgaatactttgatgctgtcaatgatgagaagaagtacaacgacagactcctgaagctgctgtgcgtgccctttggatactgcat
ccccaggtttaagaacctcagtattgaggccctctttgataggaatgaggcagcaaagtttgataaagaaaagaagaaagtttctggaaacagtgga
agtggcgcagctgcttacccctacgacgtgcctgactacgcctga

pUb PspCas13b + PspCas13b guide RNA was made by inserting the PspCas13b ORF between SalI and MluI in **pUb 3xFLAG MCS (No BsmBI) + PspCas13b guide RNA**:

aagaccatggattacaaagacgatgacgataagGTTAACGGTACCGAGCTCCCCGGGTTAATTAAAggtggatctaacatccccgctctggtggaaa
accagaagaagtactttggcacctacagcgtgatggccatgctgaacgctcagaccgtgctggaccacatccagaaggtggccgatattgagggcga
gcagaacgagaacaacgagaatctgtggtttcaccccgtgatgagccacctgtacaacgccaagaacggctacgacaagcagcccgagaaaaccatg
ttcatcatcgagcggctgcagagctacttcccattcctgaagatcatggccgagaaccagagagagtacagcaacggcaagtacaagcagaaccgcg
tggaagtgaacagcaacgacatcttcgaggtgctgaagcgcgccttcggcgtgctgaagatgtacagggacctgaccaaccactacaagacctacga
ggaaaagctgaacgacggctgcgagttcctgaccagcacagagcaacctctgagcggcatgatcaacaactactacacagtggccctgcggaacatg
aacgagagatacggctacaagacagaggacctggccttcatccaggacaagcggttcaagttcgtgaaggacgcctacggcaagaaaaagtcccaag
tgaataccggattcttcctgagcctgcaggactacaacggcgacacacagaagaagctgcacctgagcggagtgggaatcgccctgctgatctgcct
gttcctggacaagcagtacatcaacatctttctgagcaggctgcccatcttctccagctacaatgcccagagcgaggaacggcggatcatcatcaga
tccttcggcatcaacagcatcaagctgcccaaggaccggatccacagcgagaagtccaacaagagcgtggccatggatatgctcaacgaagtgaagc
ggtgccccgacgagctgttcacaacactgtctgccgagaagcagtcccggttcagaatcatcagcgacgaccacaatgaagtgctgatgaagcggag
cagcgacagattcgtgcctctgctgctgcagtatatcgattacggcaagctgttcgaccacatcaggttccacgtgaacatgggcaagctgagatac
ctgctgaaggccgacaagacctgcatcgacggccagaccagagtcagagtgatcgagcagcccctgaacggcttcggcagactggaagaggccgaga
caatgcggaagcaagagaacggcaccttcggcaacagcggcatccggatcagagacttcgagaacatgaagcgggacgacgccaatcctgccaacta
tccctacatcgtggacacctacacacactacatcctggaaaacaacaaggtcgagatgtttatcaacgacaaagaggacagcgccccactgctgccc
gtgatcgaggatgatagatacgtggtcaagacaatccccagctgccggatgagcaccctggaaattccagccatggccttccacatgtttctgttcg
gcagcaagaaaaccgagaagctgatcgtggacgtgcacaaccggtacaagagactgttccaggccatgcagaaagaagaagtgaccgccgagaatat
cgccagcttcggaatcgccgagagcgacctgcctcagaagatcctggatctgatcagcggcaatgcccacggcaaggatgtggacgccttcatcaga
ctgaccgtggacgacatgctgaccgacaccgagcggagaatcaagagattcaaggacgaccggaagtccattcggagcgccgacaacaagatgggaa
agagaggcttcaagcagatctccacaggcaagctggccgacttcctggccaaggacatcgtgctgtttcagcccagcgtgaacgatggcgagaacaa
gatcaccggcctgaactaccggatcatgcagagcgccattgccgtgtacgatagcggcgacgattacgaggccaagcagcagttcaagctgatgttc
gagaaggcccggctgatcggcaagggcacaacagagcctcatccatttctgtacaaggtgttcgcccgcagcatccccgccaatgccgtcgagttct
acgagcgctacctgatcgagcggaagttctacctgaccggcctgtccaacgagatcaagaaaggcaacagagtggatgtgcccttcatccggcggga
ccagaacaagtggaaaacacccgccatgaagaccctgggcagaatctacagcgaggatctgcccgtggaactgcccagacagatgttcgacaatgag

atcaagtcccacctgaagtccctgccacagatggaaggcatcgacttcaacaatgccaacgtgacctatctgatcgccgagtacatgaagagagtgc
tggacgacgacttccagaccttctaccagtggaaccgcaactaccggtacatggacatgcttaagggcgagtacgacagaaagggctccctgcagca
ctgcttcaccagcgtggaagagagagaaggcctctggaaagagcgggcctccagaacagagcggtacagaaagcaggccagcaacaagatccgcagc
aaccggcagatgagaaacgccagcagcgaagagatcgagacaatcctggataagcggctgagcaacagccggaacgagtaccagaaaagcgagaaag
tgatccggcgctacagagtgcaggatgccctgctgtttctgctggccaaaaagaccctgaccgaactggccgatttcgacggcgagaggttcaaact
gaaagaaatcatgcccgacgccgagaagggaatcctgagcgagatcatgcccatgagcttcaccttcgagaaaggcggcaagaagtacaccatcacc
agcgagggcatgaagctgaagaactacggcgactctttgtgctggctagcgacaagaggatcggcaacctgctggaactcgtgggcagcgacatcg
tgtccaaagaggatatcatggaagagttcaacaaatacgaccagtgcaggcccgagatcagctccatcgtgttcaacctggaaaagtgggccttcga
cacataccccgagctgtctgccagagtggaccgggaagagaaggtggacttcaagagcatcctgaaaatcctgctgaacaacaagaacatcaacaaa
gagcagagcgacatcctgcggaagatccggaacgccttcgatcacaacaattaccccgacaaaggcgtggtggaaatcaaggccctgcctgagatcg
ccatgagcatcaagaaggcctttggggagtacgccatcatgaagggaagtggctacccctacgacgtgcctgactacgcctga

pUb dPspCas13b + PspCas13b guide RNA was made by inserting the dPspCas13b ORF (catalytic dead mutations are noted in red) between SalI and MluI in **pUb 3xFLAG MCS (No BsmBI) + PspCas13b guide RNA**:

aagaccatggattacaaagacgatgacgataagGTTAACGGTACCGAGCTCCCCGGGTTAATTAAAggtggatctAACATCCCCGCTCTGGTGGAAA
ACCAGAAGAAGTACTTTGGCACCTACAGCGTGATGGCCATGCTGAACGCTCAGACCGTGCTGGACCACATCCAGAAGGTGGCCGATATTGAGGGCGA
GCAGAACGAGAACAACGAGAATCTGTGGTTTCACCCCGTGATGAGCCACCTGTACAACGCCAAGAACGGCTACGACAAGCAGCCCGAGAAAACCATG
TTCATCATCGAGCGGCTGCAGAGCTACTTCCCATTCCTGAAGATCATGGCCGAGAACCAGAGAGAGTACAGCAACGGCAAGTACAAGCAGAACCGCG
TGGAAGTGAACAGCAACGACATCTTCGAGGTGCTGAAGCGCGCCTTCGGCGTGCTGAAGATGTACAGGGACCTGACCAAC**GCA**TACAAGACCTACGA
GGAAAAGCTGAACGACGGCTGCGAGTTCCTGACCAGCACAGAGCAACCTCTGAGCGGCATGATCAACAACTACTACACAGTGGCCCTGCGGAACATG
AACGAGAGATACGGCTACAAGACAGAGGACCTGGCCTTCATCCAGGACAAGCGGTTCAAGTTCGTGAAGGACGCCTACGGCAAGAAAAAGTCCCAAG
TGAATACCGGATTCTTCCTGAGCCTGCAGGACTACAACGGCGACACACAGAAGAAGCTGCACCTGAGCGGAGTGGGAATCGCCCTGCTGATCTGCCT
GTTCCTGGACAAGCAGTACATCAACATCTTTCTGAGCAGGCTGCCCATCTTCTCCAGCTACAATGCCCAGAGCGAGGAACGGCGGATCATCATCAGA
TCCTTCGGCATCAACAGCATCAAGCTGCCCAAGGACCGGATCCACAGCGAGAAGTCCAACAAGAGCGTGGCCATGGATATGCTCAACGAAGTGAAGC
GGTGCCCCGACGAGCTGTTCACAACACTGTCTGCCGAGAAGCAGTCCCGGTTCAGAATCATCAGCGACGACCACAATGAAGTGCTGATGAAGCGGAG
CAGCGACAGATTCGTGCCTCTGCTGCTGCAGTATATCGATTACGGCAAGCTGTTCGACCACATCAGGTTCCACGTGAACATGGGCAAGCTGAGATAC
CTGCTGAAGGCCGACAAGACCTGCATCGACGGCCAGACCAGAGTCAGAGTGATCGAGCAGCCCCTGAACGGCTTCGGCAGACTGGAAGAGGCCGAGA
CAATGCGGAAGCAAGAGAACGGCACCTTCGGCAACAGCGGCATCCGGATCAGAGACTTCGAGAACATGAAGCGGGACGACGCCAATCCTGCCAACTA
TCCCTACATCGTGGACACCTACACACACTACATCCTGGAAAACAACAAGGTCGAGATGTTTATCAACGACAAAGAGGACAGCGCCCCACTGCTGCCC
GTGATCGAGGATGATAGATACGTGGTCAAGACAATCCCCAGCTGCCGGATGAGCACCCTGGAAATTCCAGCCATGGCCTTCCACATGTTTCTGTTCG
GCAGCAAGAAAACCGAGAAGCTGATCGTGGACGTGCACAACCGGTACAAGAGACTGTTCCAGGCCATGCAGAAAGAAGAAGTGACCGCCGAGAATAT
CGCCAGCTTCGGAATCGCCGAGAGCGACCTGCCTCAGAAGATCCTGGATCTGATCAGCGGCAATGCCCACGGCAAGGATGTGGACGCCTTCATCAGA
CTGACCGTGGACGACATGCTGACCGACACCGAGCGGAGAATCAAGAGATTCAAGGACGACCGGAAGTCCATTCGGAGCGCCGACAACAAGATGGGAA
AGAGAGGCTTCAAGCAGATCTCCACAGGCAAGCTGGCCGACTTCCTGGCCAAGGACATCGTGCTGTTTCAGCCCAGCGTGAACGATGGCGAGAACAA
GATCACCGGCCTGAACTACCGGATCATGCAGAGCGCCATTGCCGTGTACGATAGCGGCGACGATTACGAGGCCAAGCAGCAGTTCAAGCTGATGTTC
GAGAAGGCCCGGCTGATCGGCAAGGGCACAACAGAGCCTCATCCATTTCTGTACAAGGTGTTCGCCCGCAGCATCCCCGCCAATGCCGTCGAGTTCT
ACGAGCGCTACCTGATCGAGCGGAAGTTCTACCTGACCGGCCTGTCCAACGAGATCAAGAAAGGCAACAGAGTGGATGTGCCCTTCATCCGGCGGGA
CCAGAACAAGTGGAAAACACCCGCCATGAAGACCCTGGGCAGAATCTACAGCGAGGATCTGCCCGTGGAACTGCCCAGACAGATGTTCGACAATGAG
ATCAAGTCCCACCTGAAGTCCCTGCCACAGATGGAAGGCATCGACTTCAACAATGCCAACGTGACCTATCTGATCGCCGAGTACATGAAGAGAGTGC
TGGACGACGACTTCCAGACCTTCTACCAGTGGAACCGCAACTACCGGTACATGGACATGCTTAAGGGCGAGTACGACAGAAAGGGCTCCCTGCAGCA
CTGCTTCACCAGCGTGGAAGAGAGAGAAGGCCTCTGGAAAGAGCGGGCCTCCAGAACAGAGCGGTACAGAAAGCAGGCCAGCAACAAGATCCGCAGC
AACCGGCAGATGAGAAACGCCAGCAGCGAAGAGATCGAGACAATCCTGGATAAGCGGCTGAGCAACAGCCGGAACGAGTACCAGAAAAGCGAGAAAG
TGATCCGGCGCTACAGAGTGCAGGATGCCCTGCTGTTTCTGCTGGCCAAAAAGACCCTGACCGAACTGGCCGATTTCGACGGCGAGAGGTTCAAACT
GAAAGAAATCATGCCCGACGCCGAGAAGGGAATCCTGAGCGAGATCATGCCCATGAGCTTCACCTTCGAGAAAGGCGGCAAGAAGTACACCATCACC
AGCGAGGGCATGAAGCTGAAGAACTACGGCGACTTCTTTGTGCTGGCTAGCGACAAGAGGATCGGCAACCTGCTGGAACTCGTGGGCAGCGACATCG
TGTCCAAAGAGGATATCATGGAAGAGTTCAACAAATACGACCAGTGCAGGCCCGAGATCAGCTCCATCGTGTTCAACCTGGAAAAGTGGGCCTTCGA
CACATACCCCGAGCTGTCTGCCAGAGTGGACCGGGAAGAGAAGGTGGACTTCAAGAGCATCCTGAAAATCCTGCTGAACAACAAGAACATCAACAAA
GAGCAGAGCGACATCCTGCGGAAGatccggaacgccttcgat**cac**aacaattaccccgacaaaggcgtggtggaaatcaaggccctgcctgagatcg
ccatgagcatcaagaaggcctttggggagtacgccatcatgaagggaagtggctacccctacgacgtgcctgactacgcctga

pUb LwaCas13a + LwaCas13a guide RNA was made by inserting the LwaCas13a ORF sequence between SpeI and AgeI in **pUb 3xFLAG MCS (No BsmBI) + LwaCas13a guide RNA**:

caaagtgaccaaggtcgacggcatcagccacaagaagtacatcgaagagggcaagctcgtgaagtccaccagcgaggaaaaccggaccagcgagaga
ctgagcgagctgctgagcatccggctggacatctacatcaagaaccccgacaacgcctccgaggaagagaaccggatcagaagagagaacctgaaga
agttctttagcaacaaggtgctgcacctgaaggacagcgtgctgtatctgaagaaccggaaagaaaagaacgccgtgcaggacaagaactatagcga
agaggacatcagcgagtacgacctgaaaaacaagaacagcttctccgtgctgaagaagatcctgctgaacgaggacgtgaactctgaggaactggaa
atctttcggaaggacgtggaagccaagctgaacaagatcaacagcctgaagtacagcttcgaagagaacaaggccaactaccagaagatcaacgaga

```
acaacgtggaaaaagtgggcggcaagagcaagcggaacatcatctacgactactacagagagagcgccaagcgcaacgactacatcaacaacgtgca
ggaagccttcgacaagctgtataagaaagaggatatcgagaaactgtttttcctgatcgagaacagcaagaagcacgagaagtacaagatccgcgag
tactatcacaagatcatcggccggaagaacgacaaagagaacttcgccaagattatctacgaagagatccagaacgtgaacaacatcaaagagctga
ttgagaagatccccgacatgtctgagctgaagaaaagccaggtgttctacaagtactacctggacaaagaggaactgaacgacaagaatattaagta
cgccttctgccacttcgtggaaatcgagatgtcccagctgctgaaaaactacgtgtacaagcggctgagcaacatcagcaacgataagatcaagcgg
atcttcgagtaccagaatctgaaaaagctgatcgaaaacaaactgctgaacaagctggacacctacgtgcggaactgcggcaagtacaactactatc
tgcaagtgggcgagatcgccacctccgactttatcgcccggaaccggcagaacgaggccttcctgagaaacatcatcggcgtgtccagcgtggccta
cttcagcctgaggaacatcctggaaaccgagaacgagaacggtatcaccggccggatgcggggcaagaccgtgaagaacaacaagggcgaagagaaa
tacgtgtccggcgaggtggacaagatctacaatgagaacaagcagaacgaagtgaaagaaaatctgaagatgttctacagctacgacttcaacatgg
acaacaagaacgagatcgaggacttcttcgccaacatcgacgaggccatcagcagcatcagacacggcatcgtgcacttcaacctggaactggaagg
caaggacatcttcgccttcaagaatatcgcccccagcgagatctccaagaagatgtttcagaacgaaatcaacgaaaagaagctgaagctgaaaatc
ttcaagcagctgaacagcgccaacgtgttcaactactacgagaaggatgtgatcatcaagtacctgaagaataccaagttcaacttcgtgaacaaaa
acatccccttcgtgcccagcttcaccaagctgtacaacaagattgaggacctgcggaataccctgaagtttttttggagcgtgcccaaggacaaaga
agagaaggacgcccagatctacctgctgaagaatatctactacggcgagttcctgaacaagttcgtgaaaaactccaaggtgttctttaagatcacc
aatgaagtgatcaagattaacaagcagcggaaccagaaaaccggccactacaagtatcagaagttcgagaacatcgagaaaaccgtgcccgtggaat
acctggccatcatccagagcagagagatgatcaacaaccaggacaaagaggaaaagaatacctacatcgactttattcagcagattttcctgaaggg
cttcatcgactacctgaacaagaacaatctgaagtatatcgagagcaacaacaacaatgacaacaacgacatcttctccaagatcaagatcaaaaag
gataacaaagagaagtacgacaagatcctgaagaactatgagaagcacaatcggaacaaagaaatccctcacgagatcaatgagttcgtgcgcgaga
tcaagctggggaagattctgaagtacaccgagaatctgaacatgttttacctgatcctgaagctgctgaaccacaaagagctgaccaacctgaaggg
cagcctggaaaagtaccagtccgccaacaaagaagaaaccttcagcgacgagttggaactgatcaacctgctgaacctggacaacaacagagtgacc
gaggacttcgagctggaagccaacgagatcggcaagttcctggacttcaacgaaaacaaaatcaaggaccggaaagagctgaaaaagttcgacacca
acaagatctatttcgacggcgagaacatcatcaagcaccgggccttctacaatatcaagaaatacggcatgctgaatctgctggaaaagatcgccga
taaggccaagtataagatcagcctgaaagaactgaaagagtacagcaacaagaagaatgagattgaaaagaactacaccatgcagcagaacctgcac
cggaagtacgccagacccaagaaggacgaaaagttcaacgacgaggactacaaagagtatgagaaggccatcggcaacatccagaagtacacccacc
tgaagaacaaggtggaattcaatgagctgaacctgctgcagggcctgctgctgaagatcctgcaccggctcgtgggctacaccagcatctgggagcg
ggacctgagattccggctgaagggcgagtttcccgagaaccactacatcgaggaaattttcaatttcgacaactccaagaatgtgaagtacaaaagc
ggccagatcgtggaaaagtatatcaacttctacaaagaactgtacaaggacaatgtggaaaagcggagcatctactccgacaagaaagtgaagaaac
tgaagcaggaaaaaaaggacctgtacatccggaactacattgcccacttcaactacatcccccacgccgagattagcctgctggaagtgctggaaaa
cctgcggaagctgctgtcctacgaccggaagctgaagaacgccatcatgaagtccatcgtggacattctgaaagaatacggcttcgtggccaccttc
aagatcggcgctgacaagaagatcgaaatccagaccctggaatcagagaagatcgtgcacctgaagaatctgaagaaaaagaaactgatgaccgacc
ggaacagcgaggaactgtgcgaactcgtgaaagtcatgttcgagtacaaggccctggaaggaagtggctacccctacgacgtgcctgactacgcctg
a
```

Guide RNA sequences were then cloned into the corresponding plasmids described above. All guide RNAs used in the study were ordered as paired forward/reverse oligos and the sequences are provided in **Supplementary Tables S1 and S2**. Oligos were phosphorylated and annealed using the following 10 μL reactions:

1 μL	100 μM Forward oligo
1 μL	100 μM Reverse oligo
1 μL	10 mM ATP
1 μL	10x T4 Polynucleotide Kinase Buffer
0.5 μL	T4 Polynucleotide Kinase (NEB)
5.5 μL	Water

Reactions were incubated in a PCR machine at 37°C for 30 min, followed by 95°C for 5 min, followed by a ramp down to 25°C (0.1°C/sec). Ligations were then performed using purified plasmids cut with BsmBI.

Drosophila expression plasmids expressing only a guide RNA (without Cas13 effector) were generated from **pUb 3xFLAG MCS (No BsmBI) + RxCas13d guide RNA** (for plasmids used in **Supplementary Figure S2**) or from **pUb 3xFLAG MCS (No BsmBI) + PspCas13b guide RNA**

(for plasmids used in **Supplementary Figure S7**) by phosphorylating, annealing, and ligating the guide RNA oligos as described above.

Reporter plasmids generated:

Hy_pMtnA mCherry SV40 (Addgene #176302) was made by inserting a 3x FLAG tagged mCherry ORF between the XhoI and NotI sites in **Hy_pMT EGFP SV40 pA Sense** (Addgene #69911):

```
accatggactacaaagaccatgacggtgattataaagatcatgacatcgattacaaggatgacgatgacaagaccggtGTGAGCAAGGGCGAGGAgg
ataacatggccatcatcaaggagttcatgcgcttcaaggtgcacatggagggctccgtgaacggccacgagttcgagatcgagggcgagggcgaggg
ccgcccctacgagggcacccagaccgccaagctgaaggtgaccaagggtggcccctgcccttcgcctgggacatcctgtcccctcagttcatgtac
ggctccaaggcctacgtgaagcaccccgccgacatccccgactacttgaagctgtccttccccgagggcttcaagtgggagcgcgtgatgaacttcg
aggacggcggcgtggtgaccgtgacccaggactcctccctgcaggacggcgagttcatctacaaggtgaagctgcgcggcaccaacttcccctccga
cggccccgtaatgcagaagaagaccatgggctgggaggcctcctccgagcggatgtaccccgaggacggcgccctgaagggcgagatcaagcagagg
ctgaagctgaaggacggcggccactacgacgctgaggtcaagaccacctacaaggccaagaagcccgtgcagctgcccggcgcctacaacgtcaaca
tcaagttggacatcacctcccacaacgaggactacaccatcgtggaacagtacgaacgcgccgagggccgccactccaccggcggCATGGACGAGCT
GTACAAGTAG
```

Hy_pMtnA FFLuc SV40 (Addgene #176299) was made by inserting the firefly luciferase (FFLuc) ORF between the XhoI and NotI sites in **Hy_pMT EGFP SV40 pA Sense** (Addgene #69911):

```
accatggaagatgccaaaaacattaagaagggcccagcgccattctacccactcgaagacgggaccgccggcgagcagctgcacaaagccatgaagc
gctacgccctggtgcccggcaccatcgcctttaccgacgcacatatcgaggtggacattacctacgccgagtacttcgagatgagcgttcggctggc
agaagctatgaagcgctatgggctgaatacaaaccatcggatcgtggtgtgcagcgagaatagcttgcagttcttcatgcccgtgttgggtgccctg
ttcatcggtgtggctgtggccccagctaacgacatctacaacgagcgcgagctgctgaacagcatgggcatcagccagcccaccgtcgtattcgtga
gcaagaaagggctgcaaaagatcctcaacgtgcaaaagaagctaccgatcatacaaaagatcatcatcatggatagcaagaccgactaccagggctt
ccaaagcatgtacaccttcgtgacttcccatttgccacccggcttcaacgagtacgacttcgtgcccgagagcttcgaccgggacaaaaccatcgcc
ctgatcatgaacagtagtggcagtaccggattgcccaagggcgtagccctaccgcaccgcaccgcttgtgtccgattcagtcatgcccgcgacccca
tcttcggcaaccagatcatccccgacaccgctatcctcagcgtggtgccatttcaccacggcttcggcatgttcaccacgctgggctacttgatctg
cggctttcgggtcgtgctcatgtaccgcttcgaggaggagctattcttgcgcagcttgcaagactataagattcaatctgccctgctggtgcccaca
ctatttagcttcttcgctaagagcactctcatcgacaagtacgacctaagcaacttgcacgagatcgccagcggcggggcgccgctcagcaaggagg
taggtgaggccgtggccaaacgcttccacctaccaggcatccgccagggctacggcctgacagaaacaaccagcgccattctgatcacccccgaagg
ggacgacaagcctggcgcagtaggcaaggtggtgcccttcttcgaggctaaggtggtggacttggacaccggtaagacactgggtgtgaaccagcgc
ggcgagctgtgcgtccgtggccccatgatcatgagcggctacgttaacaaccccgaggctacaaacgctctcatcgacaaggacggctggctgcaca
gcggcgacatcgcctactgggacgaggacgagcacttcttcatcgtggaccggctgaagagcctgatcaaatacaagggctaccaggtagccccagc
cgaactggagagcatcctgctgcaacaccccaacatcttcgacgccggggtcgccggcctgcccgacgacgatgccggcgagctgcccgccgcagtc
gtcgtgctggaacacggtaaaaccatgaccgagaaggagatcgtggactatgtggccagccaggttacaaccgccaagaagctgcgcggtggtgttg
tgttcgtggacgaggtgcctaaaggactgaccggcaagttggacgcccgcaagatccgcgagattctcattaaggccaagaagggcggcaagatcgc
cgtggactacaaagaccatgacggtgattataaagatcatgacatcgattacaaggatgacgatgacaagtaa
```

Hy_pUbi-p63e mCherry SV40 (Addgene #176300) was made by inserting the mCherry ORF between the XbaI and NotI sites in **Hy_pUbi-p63e eGFP SV40** (Addgene #132650):

```
ATGGTGAGCAAGGGCGAGGAggataacatggccatcatcaaggagttcatgcgcttcaaggtgcacatggagggctccgtgaacggccacgagttcg
agatcgagggcgagggcgagggccgcccctacgagggcacccagaccgccaagctgaaggtgaccaagggtggcccctgcccttcgcctgggacat
cctgtcccctcagttcatgtacggctccaaggcctacgtgaagcaccccgccgacatccccgactacttgaagctgtccttccccgagggcttcaag
tgggagcgcgtgatgaacttcgaggacggcggcgtggtgaccgtgacccaggactcctccctgcaggacggcgagttcatctacaaggtgaagctgc
gcggcaccaacttcccctccgacggccccgtaatgcagaagaagaccatgggctgggaggcctcctccgagcggatgtaccccgaggacggcgccct
gaagggcgagatcaagcagaggctgaagctgaaggacggcggccactacgacgctgaggtcaagaccacctacaaggccaagaagcccgtgcagctg
```

```
cccggcgcctacaacgtcaacatcaagttggacatcacctcccacaacgaggactacaccatcgtggaacagtacgaacgcgccgagggccgccact
ccaccggcggCATGGACGAGCTGTACAAGTGA
```

pcDNA3.1(+) mCherry (Addgene #176301) was made by inserting the mCherry ORF between the HindIII and XbaI sites in **pcDNA3.1(+) eGFP** (Addgene #129020):

```
GccaccATGGTGAGCAAGGGCGAGGAggataacatggccatcatcaaggagttcatgcgcttcaaggtgcacatggagggctccgtgaacggccacg
agttcgagatcgagggcgagggcgagggccgcccctacgagggcacccagaccgccaagctgaaggtgaccaagggtggccccctgcccttcgcctg
ggacatcctgtcccctcagttcatgtacggctccaaggcctacgtgaagcaccccgccgacatccccgactacttgaagctgtccttccccgagggc
ttcaagtgggagcgcgtgatgaacttcgaggacggcggcgtggtgaccgtgacccaggactcctccctgcaggacggcgagttcatctacaaggtga
agctgcgcggcaccaacttcccctccgacggccccgtaatgcagaagaagaccatgggctgggaggcctcctccgagcggatgtaccccgaggacgg
cgccctgaagggcgagatcaagcagaggctgaagctgaaggacggcggccactacgacgctgaggtcaagaccacctacaaggccaagaagcccgtg
cagctgcccggcgcctacaacgtcaacatcaagttggacatcacctcccacaacgaggactacaccatcgtggaacagtacgaacgcgccgagggcc
gccactccaccggcggCATGGACGAGCTGTACAAGTGA
```
